# Reexamining Fat: Exploring Diversity, Plasticity, Development, Functional Implication, and Therapeutic Options

**DOI:** 10.3390/ijms27041925

**Published:** 2026-02-17

**Authors:** Presley D. Dowker-Key, Praveen Kumar Jadi, Rawon Alfatlawi, Richard J. Giannone, Ahmed Bettaieb

**Affiliations:** 1 Department of Nutrition, University of Tennessee, Knoxville, TN 37996-0840, USA; 2 Department of Biomedical Engineering, University of Tennessee, Knoxville, TN 37996-0840, USA; 3 Oak Ridge National Laboratory, Oak Ridge, TN 37831-6191, USA; 4 Graduate School of Genome Science and Technology, University of Tennessee, Knoxville, TN 37996-0840, USA; 5 Department of Biochemistry, Cellular and Molecular Biology, University of Tennessee, Knoxville, TN 37996-0840, USA

**Keywords:** obesity, adipose biology, metabolism, BAT, WAT, YAT, PAT, beige, brite, browning

## Abstract

Obesity has become so prevalent in many developed countries that it is increasingly perceived as a new norm, despite decades of interventions and drug development. Although research continues to explore novel strategies, no single approach to date has demonstrated sustained success in reducing its population-level dominance. This underscores the need to better evaluate and integrate the growing body of knowledge surrounding obesity’s multifaceted nature. Stamped under one ‘fat’ name, adipose tissue varies by color, location, morphology, composition, and function. This variability suggests a level of complexity that demands deeper investigation. Although the relevance and roles of different adipose types have been extensively discussed throughout the literature, their interdependence, synergy, and collective impact on the body remain to be fully expounded. This review aims to further consolidate and elucidate the available information on the different adipose tissue types and their association with obesity and metabolic health. We also discuss existing and emerging therapeutic strategies, highlighting their respective strengths and limitations.

## 1. Introduction

Obesity is an increasing health concern that was formally recognized by the World Health Organization as a global epidemic in the late 90s [1]. Among developed countries, the United States is projected to have one of the highest obesity rates by 2030, affecting one in two American adults [2]. Obesity’s detriment is largely attributed to various complex comorbidities that accompany it’s more modest definition—excess fat accumulation that poses a threat to human health [3]. While many associated comorbidities directly affect physical health [4], obesity has also been reported to inflict economic, social, and psychological burden [5,6]. In fact, aside from direct medical costs, other aspects of livelihood, such as work productivity, transportation, and overall human capital, are severely impacted by this disease [7]. Therefore, metabolic dysfunction is just the tip of the iceberg.

Traditional approaches to determining obesity have relied heavily on body mass index (BMI) to estimate excess adiposity and related health risk. Contemporary frameworks instead support a more refined classification that distinguishes between preclinical and clinical obesity, with preclinical obesity describing excess adiposity in the setting of preserved organ and tissue function but elevated risk for future metabolic disease, and clinical obesity marked by clear organ dysfunction or functional limitations attributable to excess fat [8]. In this newer model, BMI is used primarily as a population-level surrogate rather than an individual diagnostic tool, and clinical assessment of obesity emphasizes confirmation of excess fat through direct body composition measures or validated anthropometric indices such as waist circumference and waist-to-hip ratio [8]. According to this strategy, individuals identified with preclinical obesity are directed toward structured counseling and preventive monitoring, whereas those with clinical obesity are offered evidence-based interventions aimed at improving or reversing obesity-related health impairment; this approach is consistent with the widely accepted definition of obesity as a complex, multifactorial chronic disease.

Despite this more recent consensus, BMI is still commonly used in clinical practice to determine a patient’s weight category. BMI classifications range from <18.5 kg/m^2^ (underweight) to 40 kg/m^2^ (class III obese), with the lowest mortality rate correlated with a BMI of 20.0–24.9 kg/m^2^ [9]. Although BMI is a cost-effective, straightforward measurement, it faces several limitations when used as a tool to indicate whole body adiposity and obesity. For instance, inherent bounds such as differences among race and ethnicity have been shown to influence BMI classification [10]. Moreover, the limitations associated with the full reliance on BMI to directly distinguish the body’s composition greatly reduces its ingenuity to infer fat mass or denote health status [11,12]. Therefore, other calculated measurements including waist-to-stature ratio, waist circumference, and waist-to-hip ratio have been suggested to assess body fat to account for BMI’s imprecision [8].

More advanced anthropometric tools, such as dual-energy X-ray absorptiometry, have significantly improved the accuracy of evaluating body fat; however, these measures still lack the competence to justify the core cause of obesity [13,14,15,16]. Furthermore, there are many factors that can influence an individual’s predisposition to obesity [17], although often placed at fault is a ‘simple’ energy imbalance that caters to a positive intake and negative output. The commonly referenced Western diet is notorious for being energy dense, having high sugar content, and serving sizable portions. The high caloric index of Western foods has been shown to negatively influence gut microbiome and energy metabolism and, in turn, promote obesity [18]. However, diet is considered a modifiable factor of obesity; thus, actions can be taken to alter outcomes [19]. Additionally, levels of physical activity, stress, and sleeping patterns have also been reported as modifiable factors that either promote or discourage weight gain and obesity, depending on an individual’s routine habits [20,21,22].

While the previously mentioned factors are amendable, others such as age, sex, race/ethnicity, and genetic makeup are considered non-modifiable [23]. For instance, the first identified and, still to-date, most renowned gene locus correlated with increased body weight and obesity risk is the fat mass obesity-associated gene [24]. Moreover, it has been demonstrated that ~17% of the phenotypic variance in BMI can be linked to common single-nucleotide polymorphisms [25]. In fact, a genome-wide association study suggested common variances may even account for more than 20% of BMI differences [26]. While genetics plays a recognized role, it does not fully explain the differences in BMI among individuals. Consequently, there is a growing interest in understanding the role of epigenetics and its relationship to obesity development. Epigenome-wide association studies have reported gene–environment interactions that underly the risk of obesity and metabolic diseases [27]. Moreover, a subset of endocrine disrupting chemicals, referred to as ‘obesogens’, have also been suggested to alter obesity risk [28]. The identification of these risk factors has not only extended our understanding of how obesity may be initiated or nurtured but also has unveiled several approaches that may aid in its prevention. However, a large portion of the target population is beyond the benefits of receiving preventative care, and retrospective actions are required. Therefore, characterizing pathophysiological outcomes and clinical manifestations of obesity will be critical for developing effective treatments that provide sustained benefits.

It is well-known that obesity contributes to metabolic and organ dysfunction [29]. Under normal physiological conditions, fat is traditionally stored in the form of triacylglycerol (TAG), which protects the body against damage that can be caused by oxidative stress due, in part, to excessive release and circulation of free fatty acids (FFAs) [30]. Furthermore, since obesity supports an excess accumulation of fat, lipids infiltrate and settle in less suitable tissues, leading to lipotoxicity and a variety of clinical consequences such as insulin resistance and type 2 diabetes (T2DM). In fact, the repeatedly demonstrated association between obesity and diabetes granted the now widely recognized, portmanteau “diabesity” [30,31]. Moreover, obesity increases cardiovascular disease risk factors [32] while also facilitating an inflammatory state that largely affects adipose tissue homeostasis and function [33]. For instance, obesity may disrupt immune homeostasis by elevating pro-inflammatory cytokines while suppressing anti-inflammatory mediators, driven by a pronounced shift in adipose tissue macrophage (ATM) polarization from predominantly anti-inflammatory M2-like to pro-inflammatory M1-like states [32,34,35]. In addition, pulmonary disorders and hepatic and renal diseases, as well as neurological disturbances, have been noted in many cases [36,37]. Despite the many decades of obesity research that have resulted in an extensive catalog of therapies, the ongoing search for a more effective and long-lasting option(s) highlights their struggle to efficiently mitigate the problem. Therefore, the following section will examine current obesity therapies and their limitations, aiming to provide a comprehensive overview of the current state of treatment options.

## 2. Current Obesity Therapies and Their Limitations

**Figure 1:**
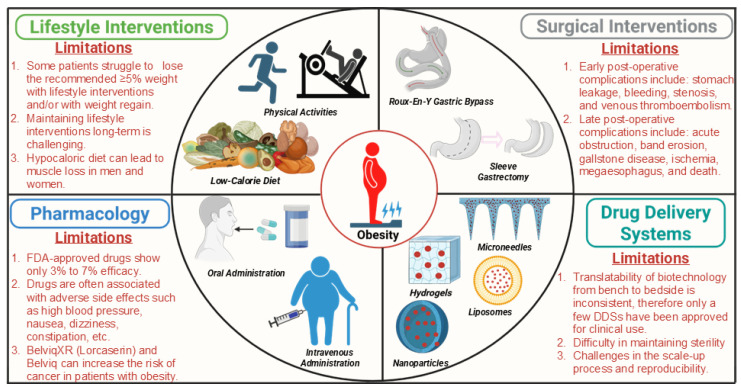
Limitations of Current Approaches to Treat Obesity. The first-line approach to obesity management involves lifestyle modifications, such as adopting low-calorie diets and increasing physical activity, which can achieve weight loss and improve health outcomes [40,41,42,43,44,45,46,47]. However, their effectiveness is often limited by adherence challenges. Pharmacotherapy is recommended for those who do not meet weight loss goals through lifestyle changes alone and is typically used in combination to enhance appetite control and satiety [48,49]. Despite its benefits, drug therapy is often constrained by side effects and safety concerns [50,51,52]. Bariatric surgery, reserved for individuals with severe obesity (BMI ≥40 kg/m^2^), can significantly improve metabolic and cardiovascular parameters but carries postoperative risks and limited eligibility [53,54]. Given these constraints, emerging strategies such as advanced DDSs aim to enhance therapeutic efficacy and minimize adverse effects, though issues of safety and efficacy remain [55]. Created in BioRender. Dowker, P. (2026) https://BioRender.com/r25o517 (Accessed on 10 February 2026). Footnotes and abbreviations: Body mass index (BMI); Drug delivery systems (DDSs); Food and Drug Administration (FDA); Lorcaserin (Belviq XR).

The heterogeneity of obesity—its varying causes, complications, and clinical presentation—presents a significant challenge for treatment [38]. Current therapeutic approaches include non-pharmacological interventions (lifestyle modifications), pharmacological options (anti-obesity drugs and gene therapy), and surgical interventions (bariatric surgery) [39] (Figure 1).

Lifestyle modifications with low-calorie diets, high levels of physical activity, and behavioral interventions have shown beneficial effects on weight loss and glycemic control in patients with obesity with T2DM [56,57,58]. However, most patients experience weight regain without continuous work. Additionally, adherence can be extremely difficult; therefore, many patients may seek a more permanent and effortless solution [59,60]. Bariatric surgery is an intervention used to treat patients with a BMI ≥ 40 kg/m^2^ (severe obesity) or BMI ≥ 35 kg/m^2^ (moderate obesity) and with at least one obesity-associated disorder [61,62,63]. Bariatric approaches are divided into malabsorptive (proximal jejunum was bypassed to the distal ileum), restricted (reduces the size of the stomach), and combination operations [60,61]. Worldwide, the most frequently used bariatric procedures are Roux-en-Y gastric bypass, (the upper part of the stomach is divided into small proximal), sleeve gastrectomy (the stomach is excised leaving a narrow medial aspect), biliopancreatic diversion with duodenal switch (sleeve gastrectomy-like gastrectomy is performed forming tubular pouch and small intestine cut in two places), and adjustable gastric banding procedures [64,65,66]. Bariatric surgeries promote weight loss and potentially improve metabolic profile in patients with obesity; however, they are associated with several complications, including infection of the gastric band, sepsis, anastomotic leaks, iron deficiency, and venous thromboembolism [61,67]. Therefore, due to high costs, specified eligibility, and accessory complications related to surgery, non-surgical interventions combined with anti-obesity drugs (AODs) may be more suitable for the common majority.

AODs initiate weight loss by reducing food intake and enhancing satiety by acting on the central nervous system, mainly on the arcuate nucleus of the hypothalamus [68]. Studies have shown that usage of AODs along with lifestyle modifications enhanced achievable weight loss [69,70]. Throughout the history of AODs, numerous drug molecules were introduced into the market for treating obesity; however, most of them were withdrawn from the market due to their adverse side effects. For example, dinitrophenol was used to treat patients with obesity in the 1930s, but it was withdrawn due to its irreversible side effects, such as rashes, cataracts, and even death due to hyperthermia [71]. Amphetamine and amphetamine-related compounds were approved in the 1940s and 1950s, yet their use became limited due to the struggle to define the efficacy and safety of these drugs and their addictions [72]. Trends of discontinuing drugs from clinical use continued until drugs like fenfluramine plus phentermine (fen-phen) and dexfenfluramine were approved in the mid-1990s, which gave rise to long-term use to treat obesity. However, adverse effects linked with fen-phen, including valvular abnormalities [73] and dexfenfluramine-induced pulmonary hypertension and neurotoxicity in preclinical models [72] caused a withdrawal from the market. FDA-approved drugs for treating obesity, their mode of action, and their side effects are described in Table 1. From the various studies that have explored new AODs, the drugs that are in clinical trials are outlined in Table 2. In addition to the chemically synthetic compounds, molecules derived from natural products such as polyphenols, flavonoids, alkaloids, etc., have been shown to have anti-obesity activity (e.g., promoting the browning of white adipose tissue (WAT)) [74]; however, their clinical relevance are limited due to their poor solubility, stability, and bioavailability [75,76]. To address these concerns, within the past decade, researchers have been focusing on developing drug delivery systems (DDSs) to enhance their bioavailability, specificity, and efficacy. To achieve more efficient (or better outcomes) DDSs, biocompatible polymers (natural and synthetic polymers) have been used in designing different formats like liposomes, and nano- and microcarrier systems to deliver drugs to target sites in a sustained-release manner and enhance their efficacy [77]. Comparatively, microneedles [78] and hydrogels [79] are typically used as carriers. Various nanocarrier systems are being utilized now to deliver AODs to target specific fat depots. Some examples include targeting WAT vasculature to stimulate angiogenesis, promoting the browning of WAT [80], and targeted disruption of adipose vasculature to reduce body weight in diet-induced obesity (DIO) mice [81]. Additionally, other systems have been used to enhance the internalization and bioavailability of drugs to promote brown-specific gene expression and reduce subcutaneous adipose tissue expansion [82,83]. DDSs designed for the delivery of AODs to treat obesity in vivo models are described in Table 3.

Because the development of obesity is multifactorial, the interplay between genetic, epigenetic, and environmental factors [84], as well as modulations in the functions of genes regulating appetite and body weight are a key focus of obesity research. Therefore, gene therapy may be another viable approach to treat obesity via delivering therapeutic genes into appropriate cells, restoring their function, and maintaining energy homeostasis [85]. For example, leptin-deficient mice treated with recombinant adenovirus expressing the mouse leptin cDNA resulted in a dramatic reduction in food intake and body weight [86]. In another study, a recombinant adeno-associated virus (rAAV) vector encoding leptin triggered enhanced non-shivering thermogenic (NST) energy expenditure in brown adipose tissue (BAT) [87]. Similarly, gene editing using clustered regularly interspaced short palindromic repeats (CRISPR) systems, transcription activator-like effectors, and zinc finger nucleases [77] have resulted in reductions in inflammation, body weight, and restored hepatic steatosis in obese mice [88]. Additionally, CRISPR-mediated gene activation (CRISPRa) of *Sim1* and *Mc4r* in haploinsufficient heterozygous mouse models reversed their obesity phenotype [89]. Although viral vector-associated delivery systems have demonstrated successful targeted delivery, they might be associated with immune response and broad viral tropism [90]. Therefore, novel approaches that will be as efficient yet mitigate undesirable side effects are highly warranted.

Given that adipose tissue in excess is the principal driver of obesity, a more comprehensive understanding of adipose tissue biology is essential to elucidate the development of innovative therapeutic strategies. In this review, we synthesize current knowledge on the heterogeneity of adipose tissue, outlining the defining characteristics and functions of each depot in both physiological and pathological contexts. Through this synthesis, we aim to integrate emerging evidence into a cohesive framework that not only deepens our understanding of adipose biology but also guides future development of targeted strategies to prevent and treat obesity.

**Table 1:** FDA-approved anti-obesity drugs used to treat obesity and their mode of action.

*Name of the Drug*	*Mode of Action*	*Adverse Side Effects*
Phentermine HCl	Reduces appetite and enhances metabolic rate [91].	Dry mouth, insomnia, dizziness, palpitations, flushing, fatigue, and constipation [92].
Orlistat	Inhibits pancreatic and gastrointestinal lipases, which block the hydrolysis of triglycerides and minimize fatty acid absorption by intestinal endothelium [93].	Various gastrointestinal side effects, along with oily stools, oily spotting, fecal urgency, fecal incontinence, hyper-defecation, and flatus with discharge [51,94].
Lorcaserin	A selective agonist of the 5-HT2C receptor, located in the central nervous system, that reduces caloric intake without affecting energy expenditure [95].	Headache, nausea, and dizziness [96]. Long-term usage increases the potential signal of increased cancers and cancer-related mortality [97].
Liraglutide [An analog of glucagon-like peptide-1 (GLP-1)]	Promotes weight loss by modulating appetite and enhancing satiety sensations [98].	Hypoglycemia, headache, nausea, fatigue, dizziness, diarrhea, vomiting, constipation, decreased appetite, dyspepsia, abdominal pain, and increased lipase [99].
Semaglutide (An analog of GLP-1)	Regulates appetite and caloric intake by targeting key neural circuits within the brain [100,101,102].	Nausea, vomiting, diarrhea, abdominal pain, abdominal distension, constipation, dyspepsia, headache, fatigue, dizziness, eructation, hypoglycemia in patients with type II diabetes, flatulence, gastroenteritis, and gastroesophageal reflux disease [100,101,102].
Phentermine/Topiramate	Phentermine is an appetite suppressant [99], whereas topiramate could minimize fat deposition either by stimulating energy expenditure or reducing food intake [103].	Dry mouth, insomnia, dizziness, paresthesia, constipation, and dysgeusia [99].
Naltrexone/Bupropion	Reduction in food craving [99,104].	Dry mouth, headache, nausea, dizziness, vomiting, and constipation [105].
Setmelanotide	Acts as an MC4R agonist [106].	Injection site reactions, skin hyperpigmentation, headache, and gastrointestinal side effects [106].

Footnotes and abbreviations: 5-hydroxytryptamine 2C (5-HT2C); Hydrochloride (HCl); Glucagon-like peptide-1 (GLP-1); Melanocortin-4 receptor (MC4R).

**Table 2:** Anti-obesity drugs in clinical investigation.

*Name of the Drug*	*Mode of Action*	*Adverse Side Effects*	*Clinical Phase Completed*
Tesomet (Tesofensine plus metoprolol)	Induce weight loss by reducing food intake [107].	Sleep disturbances, dry mouth, and headache [107].	Phase 2 trial has been completed (NCT03845075) [107].
Cotadutide/MEDI0382 (dual GLP-1/glucagon receptor (GCGR) agonist)	Induce weight loss by reducing food intake and increasing energy expenditure [108].	Gastrointestinal adverse events, including nausea and vomiting [108].	Clinical phase 2b study (NCT03235050) [109].
SAR425899 (dual GLP-1/GCGR agonist)	Induces weight loss by reducing food intake and increasing satiety and energy expenditure [110].	Gastrointestinal adverse events [110].	Multiple-ascending-dose trials (NCT02411825).
Tirzepatide (formerly LY3298176) (Glucose-dependent insulinotropic polypeptide (GIP) and GLP-1 receptor agonist)	Promotes weight loss through a dose-dependent reduction in food intake, accompanied by measurable decreases in hunger and fullness ratings as assessed by a visual analog scale [111,112,113]	Nausea, diarrhea, and vomiting [114]	Phase 3 trial (NCT04660643).
Cagrilintide (amylin analog)	Reduces weight by controlling appetite [115].	Gastrointestinal adverse events (nausea, constipation, and diarrhea) and administration-site reactions [116].	Phase 2 trial (NCT03856047).
Cagrilintide/Semaglutide	Promote weight loss through multiple mechanisms that regulate energy balance, including reduced energy intake, delayed gastric emptying, and central appetite suppression [112].	Gastrointestinal adverse events [117]	Phase 1b trial (NCT03600480).
*Arthrospira maxima* intake with physical exercise	Promote weight loss through several mechanisms, including inhibition of preadipocyte differentiation, reduction in de novo lipogenesis and triglyceride assembly, stimulation of lipolysis and fatty acid oxidation, and increased energy expenditure via thermogenic activation of BAT [118].	No adverse effects related to were observed during the study [119].	Double-blind, randomized, crossover trial (NCT02837666).
Velneperit (S-2367; type 5 neuropeptide Y receptor antagonist)	It suppressed food intake [120].	Nasopharyngitis, upper-respiratory infections, sinusitis, and headache [121].	Phase 2 trial (NCT01126970).

Footnotes and abbreviations: Brown adipose tissue (BAT); Glp-1/glucagon receptor (GCGR); Glucose-dependent insulinotropic polypeptide (GIP); Glucagon-like peptide-1 (GLP-1).

**Table 3:** Drug delivery systems used to deliver AODs for treating obesity in preclinical models.

*Type of DDS*	*Methods of Preparation, Conjugates, and Biocompatible* *Polymers*	*Drug*	In Vivo *Models*	*Advantages*	*Drawbacks*
Nanoparticles (NPs)	PLGA	Dibenzazepine (a γ-secretase inhibitor)	DIO mice [83].	Biocompatible and biodegradable [122]. The physicochemical properties of the NPs are easily tunable during the process of synthesis, which helps optimize drug loading and release. The surface chemistry of the NPs can be tuned to enable targeted drug delivery while minimizing dosage requirements and associated side effects. Enhanced drug delivery efficacy [39]	NP scale-up is difficult [39]. NPs’ size influences their biodistribution [123] Aggregation of NPs [122].
	Chitosan	α-lipoic acid (ALA) and caffeine	DIO Rats [124]		
	NPs were fabricated using soy PC, Kolliphor^®^ HS15, αTA, and N-(methylpolyoxyethylene oxycarbonyl)-1,2-distearoyl-sn-glycero-3-phosphoethanolami -ne (DSPE-PEG5000)-maleimide. Ligand was conjugated to DSPE-PEG5000 using maleimide conjugation.	*Trans*-resveratrol	DIO mice [125]		
	Prohibitin-targeting peptides were conjugated DSPE-PEG_5kDa_ using maleimide conjugation. NPs were synthesized using egg yolk PC, cholesterol, stearyl-octa arginine, and DSPE-PEG_5kDa_-Peptide.	Rosiglitazone	DIO mice [126]		
	Nanospheres were fabricated using PLGA and PVA		DIO LDLR^−/−^ mice [127]		
	NPs were synthesized using PLGA-b-PEG and PLGA-b-PEG-conjugated with targeted peptides (i.e., iRGD (CRGDK/RGPD/EC) or P3 (CKGGRAKDC)).		DIO mice [80]		
	Nanocarriers were fabricated using Tween 20, PC, poloxamer Synperonic PE/F68, Glycerol monostearate and cetyl palmitate, linseed oil Oleoylethanolamide and Phenyl alaninol oleamide-PAO	Capsaicin	DIO Albino Swiss mice [128]		
Liposomes	PS and PC.	IL-10	DIO mice [129]	Improved biocompatibility and biodegradability Low toxicity and antigenicity. Ability to deliver both polar and non-polar therapeutic molecules [39,122].	Reduced stability and increased drug leakage during storage
	Liposomes were synthesized using PC, cholesterol, and 1,2-distearoyl-sn-glycerol-3-phosphoethanolamine-N-[poly(ethylene glycol)-2000-maleimide (DSPE-PEG2000-MAL). Liposomes were conjugated with glucagon for targeted delivery.	T3	*Lep^ob^* mice [130]		Increased risk of aggregation. Allergic reactions. Increased risk of phospholipid oxidation and hydrolysis [39,122].
	Liposomes were synthesized using DSPC (phosphocholine), cholesterol, and PEG-2000 DSPE.	Tesaglitazar (PPARα/γ dual agonists)	*Lep^ob^* and DIO mice [131]		
Microneedle (MN) patches	Methacrylated hyaluronic acid was used as a base material for microneedle patches, whereas NPs loaded in the MN patches were synthesized using dextran.	Rosiglitazone	DIO mice [78]	Effective method for transdermal drug delivery. Prevents gastrointestinal degradation of drugs. Microneedle patches enable minimally invasive, patient-administered drug delivery [132].	Microneedle coatings support only minimal drug payloads. Fragmented microneedles retained in the skin represent a potential biohazard [132].
	PLGA/PLA	CL316243 (β3 adrenergic receptor agonist)	NIH mice [133]		
	HA	Caffeine	C57BL/6J mice [134]		
	Capsaicin-loaded α-lactalbumin nano micelles were delivered to the adipose tissue using HA MN patches.	Capsaicin	DIO mice [135]		
Hydrogels	PLGA	Epigallocatechin gallate	DIO mice [136]	Polymers facilitate controlled, sustained drug release through their inherent biocompatibility and biodegradability. Injectable hydrogels provide minimally invasive administration [137,138,139].	Low mechanical strength results in uncontrolled, rapid drug release. The hydrophilic properties restrict hydrophobic drug encapsulation and delivery. Lack of polymer and drug interactions [140,141].

Footnotes and abbreviations: α-lipoic acid (ALA); Diet-induced obesity (DIO); Interleukin (IL); Low-density lipoprotein receptor (LDLR); Nanoparticles (NPs); Phenylalaninol oleamide (PAO); Phosphatidylcholine (PC); poly(lactide-co-glycolide) (PLGA); Peroxisome proliferator-activated receptor gamma (PPARγ); Phosphatidylserine (PS); polyvinyl alcohol (PVA); alpha-tocopherol acetate (αTA).

## 3. Adipose Tissue: Distribution and Types

Adipose tissue is a specialized organ found in all mammals, as well as some non-mammalian species [142,143]. In mammals, adipose tissue congregates into well-defined regions referred to as fat depots and is dispersed across and within various parts of the body. Adipose is generally categorized into two major depots based on their anatomical location: subcutaneous (surface, underneath the skin) and visceral (deeper, surrounding or within the main body cavities) [144]. In small mammals like rodents, a commonly used preclinical model of obesity research [145], anterior subcutaneous fat includes the inter-/subscapular, superficial cervical, and axillo-thoracic regions, while posterior subcutaneous fat is localized in the inguinal, gluteal, and dorso-lumbar areas. Meanwhile, visceral adipose is found within the abdominal cavities and thorax of these mammals [143]. Conversely, BAT in rodents includes the cervical, perirenal, supraclavicular, paravertebral, axillary, and inter/intrascapular depots [145,146]. In comparison, WAT distribution in humans also spans across the body and includes the two major depots previously discussed: subcutaneous (intra-muscular, abdominal, gluteoformal) and visceral (mesenteric, gonadal, pericardial, retroperitoneal, and omental) [147]. However, BAT tends to be less static in humans. During infancy, BAT resides in the intrascapular (main depot), cervical, and perirenal regions, then later regresses and forms in the cervical, supraclavlicar, axillary, and along the spinal column [146]. Over the course of early development, BAT is regarded highly for its crucial role in core-body temperature maintenance. However, beyond infancy, it was long thought that BAT offered little to no significance in adulthood. This assumption has since been reconsidered over the last two decades as several studies have identified the presence of active BAT in adults [148,149,150]. Adult BAT is allocated both viscerally and subcutaneously. Visceral brown fat is periviscus, perivascular, or found surrounding solid organs, while subcutaneous brown fat is positioned beneath the clavicles, within the axilla and anterior abdominal wall, and between the supraclavicular fossa and anterior cervical muscles [151]. Each depot’s phenotype is primarily reflected by the ratio of white to brown adipocytes; however, a unique phenomenon known as “browning,” or “beigeing,” the conversion of white adipocytes to brown-like adipocytes (also referred to as beige, brite, or taupe), underscores the organ’s morphological plasticity and potential to alter its native function [152,153].

White adipocytes are distinguished by their voluminous unilocular lipid droplet and compressed nucleus with low mitochondrial content. On the other hand, brown adipocytes maintain smaller, multilocular lipids with substantially larger mitochondria that are abundant and equipped with the brown hallmark protein, uncoupling protein 1 (UCP1). UCP1 permits the uncoupling of oxidative phosphorylation, thus allowing for heat dissipation and characterization of BAT’s thermogenic capacity [152,153]. Conversely, WAT does not maintain the same thermogenic integrity and instead serves as a major site for energy storage [153]. Importantly, the emergence of brown-like adipocytes, often referred to as beige/brite, basally resemble and reside within WAT, yet respond to external stimuli that promote the induction of classical thermogenic gene markers [154]. This observed phenomenon, combined with its known associated metabolic benefits, has continued the renewed interest in BAT and of browning as a therapeutic approach for obesity and its associated metabolic disorders. Furthermore, two additional types have been acknowledged in the literature, although far less characterized, pink and yellow. Briefly, pink adipocytes, which arise by the transformation process of “pinking,” convert white adipocytes in the mammary glands into milk-producing/secreting epithelial cells during pregnancy and lactation [155]. Yellow adipose tissue (YAT) has been described as a fat pad that resides within the bone marrow, a depot that has both WAT and BAT characteristics, and, further, is speculated to play a role in systemic energy metabolism [156,157]. A precise characterization of the various adipose tissue types—both independently and in terms of their potential interdependence—will undoubtedly transform our understanding of the adipose organ as a whole. Accordingly, the following sections of this review will examine each adipose tissue type and its contribution to metabolic homeostasis in both health and disease.

## 4. White Adipose Tissue (WAT)

### 4.1. Energy Metabolism

The reputation of white adipose tissue has widely varied, spanning from an early write-off as an inert storage vessel to its more iniquitous interpretation as the central driver behind the obesity epidemic. However, through decades of research, WAT is now acknowledged as an essential metabolic headquarters that integrates a variety of physiological processes to support whole-body homeostasis. Therefore, in order to avoid historical faux pas, in this section, we will attempt to cover all key attributes of WAT, beginning with its earliest recognition—as an energy reservoir.

A remarkable feature of adipose tissue in general is its ability to expand. White adipocytes, in particular, are proficient, yet variable expansion artists as their diameters have been recorded from low 20 µm to upper 100 µm. This fluctuation has been associated with BMI, energy balance, depot/location and may be affected by certain metabolic diseases such as obesity and diabetes [158,159,160]. Notably, adipose tissue normally expands by either hyperplasia, hypertrophy, or both, and although each is a distinct mechanism, both processes remodel white adipocytes’ major organelle, the lipid droplet [161]. While lipid droplets vary in size across cell types, they share a common architecture: a heterogeneous interior of neutral lipids encased by a phospholipid monolayer that is associated with various proteins [162].

In white adipocytes, lipid content primarily resides as molecules of TAG, which are assembled in the last steps of de novo lipogenesis (DNL) and involve the activities of four acyltransferases to esterify free fatty acids to a glycerol backbone [163,164]. Modulation of these adipose acyltransferases has been shown to impact adipocyte development, WAT’s ability to store lipid, and overall energy metabolism [165,166]. For instance, mice deficient of glycerol-3-phosphate acyltransferase (GPAT) 3, the first enzyme of DNL, exhibited increased energy expenditure, lower body adiposity, and resistance to diet-induced weight gain [167]. However, these outcomes were expected, as previous in vitro work had shown that GPAT3 helps facilitate adipocyte differentiation and GPAT3 knockdown hinders the expression of genes related to fuel uptake and lipogenesis [168]. In line with these findings, Sim and colleagues demonstrated that GPAT3 moderately promotes 3T3-L1 adipocyte differentiation through its co-interactions with the subsequent 1-acylglycerol-3-phosphate-O-acyltransferase (AGPAT) 2 and its scaffolding protein, seipin [169].Thus, disturbances in this signaling pathway could impair normal adipocyte differentiation, leading to pathological conditions such as lipodystrophy.

Notably, mutations of either are notoriously linked to congenital generalized lipodystrophy, an autosomal recessive disorder characterized by the extreme lack of body fat and manifestation of metabolic disturbances [170]. In patients with lipodystrophy, lipids are redirected to other organs, including the liver and skeletal muscle, provoking the development of clinical abnormalities, such as non-alcoholic fatty liver disease, steatohepatitis, and insulin resistance [171,172,173]. Congenital generalized lipodystrophy emphasizes the importance of WAT lipid storage to whole-body homeostasis. Without an energy safe zone, nomadic lipids will be forcibly redirected to organs that are less equipped to handle their accumulation. Phosphatidic acid, the yield of AGPAT, is dephosphorylated by the magnesium-dependent phosphatidate phosphatase lipin 1 to form the last intermediate before TAG formation [174]. Notably, regulating the expression of lipin-1 can adopt either extreme of adiposity (obesity or lipodystrophy) [175]. For instance, while lipin-1-deficient mice fail to appropriately store TAG in adipose depots and are resistant to diet-induced obesity [176,177],, transgenic mice overexpressing lipin-1 exhibit accelerated weight gain and increased fat mass compared to wild-type mice fed the same high-fat diet [175]. In another transgenic model, adipose-specific overexpression of lipin-1 protected mice from hepatic injury induced by alcohol consumption [178]. Plausibly, overexpression of lipin-1 restored DNL and inhibited alcohol-induced lipolysis in WAT [178]. However, it was recently proposed that changes in lipin-1 expression may be less influential in humans. For instance, patients with significantly less lipin-1 exhibit less PAP activity; however, WAT development and lipid storage composition were normal [179]. Unexpectedly, others have reported that human adipose lipin-1 expression does correlate with metabolic parameters such as insulin sensitivity and BMI status [177,180,181]. Altogether, these findings suggest that additional factors may influence the discrepancies observed between rodent and human data with respect to the role of lipin-1 in energy metabolism.

DNL concludes with the activity of acyl-coenzyme A: diacylglycerol transferase (DGAT) and the formation of a TAG molecule [182]. Mammals possess two isoforms of DGAT: DGAT1 and DGAT2. Harris et al. demonstrated that adipocytes with a single deletion of either isoform were still able to sufficiently synthesize TAG and form lipid droplets, yet with a double knockout (KO) approach, synthesis activity was significantly reduced, and cells were severely devoid of lipid content [183]. Intriguingly, double KO macrophages were able to still form sterol ester-containing lipid droplets in the presence of cholesterol-rich lipoproteins, indicating that other mammalian cells may not be as heavily reliant on DGAT for lipid storage as adipocytes [183]. Although some studies concur that DGAT1 and 2 may compensate for one another upon each other’s absence, distinctive roles independent of lipid storage have also been reported [183,184,185]. For example, DGAT1 was demonstrated to play a protective role in averting adipocyte ER stress and lipotoxicity in response to high-fat feeding or stimulated lipolysis [185,186]. DGAT2 was shown to profoundly affect basal triglyceride metabolism, energy and cutaneous homeostasis, and postnatal survival [184]. On the other hand, whole-body *Dgat1* knockout mice are viable, maintain triglyceride biosynthesis, show increased energy expenditure, and exhibit enhanced insulin and leptin sensitivity [187,188,189]. Hence, DGAT1 and 2 might exhibit compensatory behavior occasionally but are by no means completely monotonous. In light of the studies above of the dynamic nature of WAT, lipid storage stands out as a vital gatekeeper of physiological homeostasis, challenging its historical reputation for being inert.

By nature, achieving any type of “balance” involves a counter; therefore, lipid storage is only half the narrative. WAT lipolysis involves the functioning of the key enzymes hormone-sensitive lipase (HSL) and adipose triglyceride lipase (ATGL), as they account for more than 95% of TG hydrolase activity in WAT [190]. It is well established that nutritional status, neuroendocrine inputs, and transcriptional regulators can influence the activities of HSL and ATGL largely by governing their intracellular localization and protein–protein interactions [191,192,193]. Briefly, canonical lipolysis begins with ATGL hydrolyzing TAG to yield an FFA and diacylglycerol (DAG). HSL then cleaves DAG to produce monoacylglycerol and another FFA. Subsequently, monoacylglycerol lipase breaks the remaining ester bond to release the last FFA from the glycerol backbone. FFAs then circulate around the body and are delivered to tissues for energy utilization. Importantly, intermediates of this process—DAG, monoacylglycerols, and FFAs—are also used as signaling messengers in various metabolic processes [194].

However, like lipogenesis, the regulation of HSL or ATGL expression impacts the efficiency of this counterbalance to maintain energy metabolism. *Atgl* whole-body-knockout mice exhibit significantly reduced lipolytic activity, mild hypertrophic obesity, and increased TG deposition in the heart, leading to cardiac dysfunction [195]. Consistently, primary triglyceride deposit cardiomyovasculopathy is characterized by mutations in the ATGL-encoding gene, *PNPLA2*, where ATGL deficiency fosters energy dysregulation and lipotoxicity of the myocardium and vasculature, consequently leading to heart failure in humans [196,197]. In an adipose-specific *Atgl*-KO model, basal lipolysis was significantly reduced, and when stimulated with a 48 h fast, mice became lethargic and hypothermic. These manifestations were arguably due to the lack of available substrate for energy production [198]. Likewise, HSL-deficient mice exhibit defective lipolytic activity and excess DAG accumulation yet seem to be resistant to genetic or diet-induced obesity [199,200,201]. But how can the consequences of one’s deficiency yield nearly opposite results to the other’s, when both enzymes appear to converge on the same pathway?

Whereas ATGL deficiency blocks the initial step of TAG hydrolysis, leading to fat entrapment, energy shortage, and lipid accumulation that promotes adipose expansion, HSL deficiency triggers a distinct compensatory response. Although HSL loss hinders lipolytic activity, it also alters the expression of genes governing adipogenic (e.g., peroxisome proliferator-activated receptor gamma (*Pparg*)) and lipogenic metabolism (e.g., *Gpat3*, *Dgat1*, *Dgat2*) in WAT [200,201], partly through the loss of intrinsic ligands required for PPARγ activation [202]. Consequently, HSL ablation not only disrupts lipid mobilization but also impairs adipocyte storage and hyperplasia—processes that can drive WAT expansion and obesity. However, as previously mentioned, when WAT fails to safely store lipid, excess FFAs are diverted to peripheral tissues, promoting ectopic fat accumulation and metabolic stress. Proper regulation of lipogenic and lipolytic enzymes is therefore essential not only for maintaining energy homeostasis but also for preserving WAT’s systemic influence, such as through its endocrine function.

### 4.2. Endocrine Function

#### 4.2.1. Leptin

It has been thirty years since the identification of the secretory factor, leptin [203]. Leptin’s mid-90s debut primed WAT’s now-accepted role as an active endocrine organ, broadening its functional repertoire. However, studies 40 years prior to the leptin era helped pave the way for its present-day recognition [204,205]. Since leptin’s discovery, decades of research have brought to light its involvement in a wide span of physiological processes, including, but not limited to, appetite control, energy metabolism, growth and development, immune support, inflammation, and bone health [206,207]. For instance, case–control studies have shown that serum leptin levels are significantly elevated in patients with rheumatoid arthritis, an inflammatory autoimmune disease moderately associated with obesity [208,209]. Leptin promotes the secretion of pro-inflammatory cytokines, which in turn enhance leptin release from the adipose tissue, establishing a positive, yet unfortunate, feedback loop [210]. However, beyond its usual roles, some evidence suggests leptin may even be able to predict the future… *well, sort of*. Some studies suggest that leptin is critically involved during postnatal development and that ensuring this hormone’s sufficiency can predispose individuals to a healthy phenotype. This discrete susceptibility to a ‘healthier, happier’ adulthood is a concept being referred to, more specifically in this setting, as nutritional or metabolic programming [211,212]. Early-life nutrition experts have suggested that breastfed infants are at a lower risk to develop childhood obesity in comparison to non-breastfed babies [213,214]. Human breast milk is known to contain a variety of essential nutrients, among them the neurotrophic leptin [215]. However, this curated confidence that ‘breast is best’ is frequently challenged, given the inherent complexity of infant/child development and maternal variability [211]. Preclinical trials remain just as perplexed due to their own varied results. Some rodent studies have indicated that the majority of neonate leptin is exogenously and maternally acquired [216] and oral administration of leptin exhibits both short-term and long-term benefits in regard to a balanced caloric intake and adult bodyweight [217,218]. In fact, murine neonates supplemented with leptin during the lactation period were shown to be protected from diet-induced adult obesity and fat accumulation [218]. Alternatively, in a recent report, the attenuation of leptin improved adult metabolism of overnourished neonate mice [219]. Notably, in cases of obesity or diabetes, it is well-established that levels of adipokines (e.g., leptin, resistin, visfatin) are elevated, which can set the stage for leptin resistance [220]. It has been further observed that leptin antagonism enhanced hypothalamic leptin sensitivity [220], implying that metabolic programming is just as reliant on proper signaling and reception as substrate availability. Therefore, breast milk’s role as nature’s cure to obesity remains to be determined. Although leptin research is emphasized, white adipose tissue is known to supply the body with an abundance of other adipose-derived hormones, consequently termed ‘adipokines’ that regulate various aspects of normal physiology.

#### 4.2.2. Adiponectin

The discovery of adiponectin closely followed that of leptin [221]. The main physiological function of this adipokine involves its relationship with glucose metabolism and insulin sensitivity [222]. These associations were first highlighted by research conducted in the early 2000s, when Berg et al. found that injecting mice with adiponectin was able to lower blood glucose in wild-type and diabetic mouse models independent of insulin [223]. Similarly, physiological doses of adiponectin, in combination with leptin, fully reversed insulin resistance in lipodystrophic mice while independent dosing of each adipokine was only partially successful [224]. These preliminary discoveries not only sparked a new era of enthusiasm in diabetic research but also propelled the exploration of adiponectin’s potential in other areas of homeostasis. Right on track and three decades later, adiponectin has been investigated in regard to both pro- and anti-inflammatory processes [225], metabolic reprogramming in cancer [226], lipid metabolism, and considered as a prospective therapeutic target for cardiovascular health [227,228].

Because adiponectin levels fluctuate with metabolic state, including obesity, it is often used as a biomarker of metabolic dysfunction [229]. Accordingly, adiponectin is notably reduced in individuals with T2DM [230] and cardiometabolic disorders [231]. Furthermore, therapies used to treat obesity-related diseases, including those mentioned above, have targeted adiponectin regulation and/or signaling mechanisms [232]. However, higher concentrations do not necessarily translate to better outcomes. The adiponectin paradox describes cases, particularly in cardiovascular disease, where elevated circulating levels of both adiponectin and leptin correlate with adverse outcomes rather than the anticipated protective effects. Impaired hepatic or renal clearance and diminished adipose tissue quality may contribute to these elevated levels without the expected metabolic benefits [233]. Therefore, attributing fixed outcomes to specific adipokines is overly simplistic, as even those traditionally viewed as beneficial, such as adiponectin, should be interpreted within their physiological context and on a case-by-case basis.

#### 4.2.3. Resistin

Resistin, the adipokine with the largest contrast to adiponectin’s metabolic effects, is a polypeptide hormone secreted from visceral obese adipose tissue [234]. In rodent models, resistin levels are inversely correlated with tissue insulin sensitivity and overall metabolic function [235,236]. Experimentally induced hyper-resistinemia causes hepatic insulin resistance and dysregulated glucose production [236,237], while transgenic overexpression impairs glucose metabolism in skeletal muscle [238]. However, as with all great scientific breakthroughs, the key consideration is clinical translatability. Therefore, when translating these findings to humans, resistin is interestingly produced by macrophages within adipose tissue rather than the adipocytes themselves [239,240]. Human resistin is strongly influenced by inflammatory stimuli, which elevate resistin and upregulate pro-inflammatory cytokines, positioning it as an active modulator of inflammation [241,242,243]. Despite this difference in cellular origin, macrophages are intrinsic components of adipose tissue as a whole [244], and resistin levels remain correlated with obesity in both humans and rodents [245] supporting its potential as a target for therapeutic or preventative strategies against metabolic disease.

#### 4.2.4. Omentin-1

Omentin, an adipokine named for its central adipose depot specificity [246], contrasts with resistin by exerting anti-inflammatory effects [247]. Circulating omentin levels are reduced in obesity and diabetes [248,249], and low omentin-1 has been identified as a potential predictor of gestational diabetes and T2DM [250]. The COVID-19 pandemic has highlighted the relevance of anti-inflammatory adipokines in disease outcomes. As such, patients with SARS-CoV-2 infection exhibit reduced serum omentin and chemerin levels, likely reflecting the heightened inflammatory state and the loss of their protective functions [251]. Independent of infection, omentin supports cardiovascular health through the promotion of vasodilation, endothelial protection, and anti-atherogenic effects [252]; a critical consideration given COVID-19’s association with long-term cardiovascular risk [253]. Therefore, omentin may be critical to both the onset and progression of obesity-related complications, including the heightened vulnerability to diseases such as COVID-19.

#### 4.2.5. The Adipose Secretome Beyond Adipokines

Adipokines have been widely studied in both health and disease, as evidenced by decades of comprehensive reviews [254,255,256]. In fact, according to Kirichenko et al., over 600 adipokines have been identified as of 2022, which has given the field invaluable insight into their roles within and outside of the adipose organ as well as whole-body metabolism [257]. However, it is well-known that WAT’s secretome extends beyond peptide-based hormones [258]. For example, lipokines are bioactive molecules secreted by the adipose tissue and act as signaling messengers across the body. Cao et al. demonstrated that C16:1n7-palmitoleate regulates systemic glucose metabolism by acting on the muscle as an insulin-sensitizing hormone [259]. More recently, a longitudinal analysis also determined that plasma palmitoleate plays a beneficial role in glucose homeostasis and insulin sensitivity in humans by cross-communicating with liver and pancreatic β-cells [260]. Other lipokines, such as palmitic acid hydroxy stearic acids (PAHSAs), particularly 5-PAHSA and 9-PAHSA, possess anti-inflammatory and anti-diabetic effects due to their ability to regulate insulin action and glucose transport [261,262]. It has been theorized that these endogenous lipids may serve as a new avenue to treat various metabolic and immune disorders, although some reports suggest otherwise [263].

Moreover, investigating the mechanisms of biomolecule transport may be just as important as studying their roles in health and disease. For example, adipose-derived extracellular vesicles (AD-EVs) have become clinically relevant due to several of their innate properties, such as low immunogenicity and biocompatibility. As such, these exosomes have been leveraged in translational medicine applications, including skin grafting, wound healing, and drug delivery [264,265]. In addition, AD-EVs have also been implicated as biomarkers for several metabolic disorders, including obesity, T2DM, cardiometabolic disease, insulin resistance, and various cancers [266]. Studies elucidating the potential role of AD-EVs in cancer progression have noted a dual function that is dependent on the cargo they deliver. For instance, while some miRNAs packaged inside may promote tumor progression and malignancy, others have been shown to have protective anti-tumor effects [267]. Furthermore, owing to the intrinsic role of EVs in intercellular communication, cancer outcomes, such as cancer cachexia, have been shown to be influenced by crosstalk mediated by extracellular vesicles exchanged between adipose and tumor tissues [268]. Clearly, WAT exhibits a highly dynamic endocrine function, and advancing therapeutic strategies for metabolic disease will require understanding not only the various biomolecules it secretes but also how they are packaged, trafficked, and received by target tissues.

### 4.3. Additional WAT Functions

In addition to energy metabolism and endocrine function, WAT has been demonstrated to serve several non-metabolic roles. For instance, dermal white adipose tissue (dWAT), a distinct category of the subcutaneous white adipose [269,270,271] found in mice and humans, provides mechanical support/cushioning, insulation, and immunity. The crude assumption that dWAT is closely related to cutaneous function and homeostasis is not necessarily overreaching. As such, dermal adipose has been shown to be involved in thermoregulation in a variety of ways. Mice with a genetic depletion of syndecan-1, a heparan sulfate proteoglycan important during tissue regeneration, were shown to exhibit an abnormal phenotype that lacked intradermal fat. Subsequently, these mutant mice were chronically cold-stressed and had increased expression of BAT markers (UCP1 and p38-α) even at thermoneutrality [272,273]. Additionally, dWAT of mice subjected to an environmental challenge or genetic manipulation was shown to thicken in order to preserve heat and maintain body temperature [274]. Interestingly, a rapid expansion of the dermal fat layer was also observed in response to a microbial infection by Staphylococcus aureus, thus indicating a plausible role for dWAT in immune response [275].

Skin is largely known to provide a protective interface that defends vital internal tissues and organs from the external environment. However, upon injury, the integumentary system is also responsible for conducting a proper immune response that supports wound healing and repair [276,277]. Dermal adipocytes have been demonstrated to contribute to wound healing of the skin by activating essential inflammatory responses such as macrophage recruitment during the early phases of repair. Moreover, the lipolytic activity of dermal adipocytes was found to influence macrophage abundance and infiltration efficiency at the site of injury [278]. This is further supported by evidence exhibited by AZIP mutant mice. Besides the classical metabolic impairment presented by AZIP mutants, Schmidt and Horsley reported that these mice, which are deficient in mature white adipocytes, including in dWAT, exhibited defective wound healing and dermal remodeling processes through altered recruitment of fibroblasts to the wound bed [279]. Therefore, dWAT is a distinct subset of white adipocytes that highlights WAT’s role in providing mechanical support and immune health, complementing its more commonly recognized functions in metabolism and hormonal secretion.

Overall, WAT is not inherently harmful to the body. In fact, WAT accounts for roughly one-fourth of a healthy individual’s total body weight and is clearly necessary for normal physiological function. However, it is also undeniable that WAT in excess, such as what is seen in overnutrition like obesity, can lead to metabolic stress and dysfunction. Even individuals with excess adiposity in the ‘right places,’ conventionally referenced as metabolically healthy obesity, and who may be of normal weight, have been shown to have a higher cardiometabolic risk [280]. Therefore, strategies to eliminate excess WAT are warranted, with the field exploring WAT’s intrinsic plasticity as a means to reverse excess WAT accumulation.

## 5. Brown Adipose Tissue (BAT)

### 5.1. Thermoregulation

Although the renewed interest in BAT has largely stemmed from global concerns over obesity and its recognized therapeutic potential, this focus often overshadows some of BAT’s more fundamental value. Historically, brown adipose tissue was first described in the mid-16th century by Conrad Gessner, who termed it the glandula hibernica (“hibernating gland”) based on its anatomical localization in the interscapular region of hibernating marmots (*Marmota marmota*). Subsequently, this fat depot has been referred to by several other names, including the oil gland, the organ of hibernation, and, notably, brown-colored fat because of its characteristic pigmentation [281,282,283]. In a sense, hibernation is nature responding to ‘fight,’ versus ‘flight’ (e.g., migration), under severe thermal challenges, and centuries following Gessner’s interpretations, BAT was recognized as a thermogenic organ that aids in many inherent processes such as the hibernator’s torpor–arousal cycles [282,283]. Although hibernation is not pertinent to humans, the core principle remains—that being thermoregulation, or simply put, maintaining core-body temperature. The human body is programmed to be maintained at ~37 °C, give or take 0.5 degrees. Thermoreceptors across the body will communicate with the hypothalamus in order to integrate information and elicit an appropriate response [284]. Upon cold challenge, adult humans primarily rely on rapid, involuntary contractions of the skeletal muscle (shivering thermogenesis) to produce heat and reestablish temperature homeostasis, a luxury that newborns cannot yet rely on [285]. Instead, neonates are dependent on the non-shivering thermogenic capacity of BAT to adapt to the extrauterine environment [286]. BAT NST occurs by a neurophysiological mechanism that begins with the recognition of a stimulus. After birth, without corrective measures, newborns are at risk of hypothermia due to evaporative heat loss through their skin [287,288,289]. This immediate and continued loss of heat over the course of the infant’s first weeks of life initiates physiological responses mediated by hypothalamic neural signaling, which prompts sympathetic activity in BAT [290,291]. BAT receives information and direction from the output of sympathetic fibers, such as norepinephrine. From a more ‘topical’ perspective, one of BAT’s primary characteristics is its extensive innervation and vascularity. This high innervation functions as a critical component of the NST regime, which is evident based on BAT denervation studies that report that unilateral denervation greatly diminishes or completely blocks typical cold-induced effects [292]. Norepinephrine binding to β3-adrenergic receptors causes intracellular biochemical pathways such as protein kinase A- cyclic adenosine monophosphate (PKA-cAMP) to become activated and increase lipolysis for substrate availability [293]. Additionally, elevation of cAMP induces other signaling cascades such as the p38 MAPK pathway, which phosphorylates nuclear factor family members, namely peroxisome proliferator-activated receptor gamma coactivator 1-alpha (PGC-1α) and activating transcription factor 2, to transactivate the *Ucp1* promoter [294]. Ultimately, these effects are funneled down to a single organelle: the mitochondria. BAT is known to be enriched with mitochondria that are equipped with UCP1, which will recycle protons back into the matrix to generate heat at the expense of the energy equivalent, adenosine triphosphate [295,296]. Over the years, four proposed models have attempted to explain how UCP1 facilitates proton leakage: (1) H+ channel, (2) OH- channel, (3) H+ buffering model, and (4) fatty acid-cycling model. Unfortunately, due to the lack of a direct method to study UCP1 function, the exact mechanism remained elusive [297]. However, a research group from the University of California, San Francisco, utilized a patch-clamp method to directly measure proton currents across the inner mitochondrial membrane of BAT mitochondria. Their patch-clamp analysis suggested that UCP1 acts as a long-chain fatty acid (LCFA)–/H+ symporter that simultaneously transports LCFA anions and H+ across the inner membrane. LCFA anions were determined as the principal substrates of UCP1, while H+ are dependent on LCFA anions, and their pKa, to be shuttled from one side to the next [298]. This proposed shuttling mechanism offers a convincing model for how UCP1 transforms the proton motive force into heat and, moreover, how newborns defend their body temperature postpartum [299]. Of note, additional NST mechanisms that are UCP1-independent have been summarized elsewhere [300,301]; although, briefly, the notion of UCP1’s dispensability in adipose was highlighted by the peculiar findings of the Kozak group, who demonstrated that *Ucp1* KO mice on a hybrid background were able to tolerate cold exposure with gradual acclimation [302]. While many studies have corroborated the previous findings, others have gone on to identify Ca^2+^-cycling and creatine substrate-cycling pathways as thermogenic UCP1-independent mechanisms [285,300,303]. In fact, some studies have suggested that sarcolipin-based thermogenesis in the skeletal muscle may be able to compensate for the loss and/or impairment of BAT function [304,305].

### 5.2. Secretion

While WAT has been established as a major secreting organ of the body, it is becoming more apparent that BAT may also possess a secretome that can influence (i) itself (autocrine), (ii) local/surrounding (paracrine) tissues, and/or (iii) more distant (endocrine) target sites [306,307]. The community often has a candid sense of humor when it comes to nomenclature, and the naming of BAT-derived factors was no exception. Therefore, molecules secreted from brown fat that regulate metabolism are commonly referred to as ‘batokines’ [307]. Despite this standard definition, there is ongoing debate about the exact criteria for a molecule to qualify as a true batokine. This dispute has been more thoroughly addressed elsewhere [306]. However, briefly, several discussions regarding this topic have included [i] the type of biomolecule (e.g., peptide v. non-peptide); [ii] brown fat exclusivity; [iii] preferential secretion (e.g., in comparison to specifically WAT); and [iv] explicit activated/stimulated release [306,308]. Due to this discordance, all relevant studies in this section will have some mention of their overall experimental design. Moreover, in recent decades, the field has rapidly identified numerous batokine candidates, with extensive coverage provided in other reviews [307,308,309]. While batokines are peripheral to our main focus, they are redefining the landscape of brown adipose tissue biology, revealing an unexpected control over its development, activity, and thermogenic capacity.

#### 5.2.1. FGF21

Of BAT’s numerous secretory regulators, fibroblast growth factor 21 (FGF21) was one of the first to be recognized within this context. Although FGF21 is predominantly secreted by the liver [310], some studies have shown that cold exposure or adrenergic stimuli can induce the expression of FGF21 in other tissues, including adipose tissue [311]. For instance, around the time of BAT’s functional (re)discovery [148,149,312], Chartoumpekis et al. found that short-term cold exposure and/or adrenergic stimulation increases the expression of FGF21 in BAT but not in the liver. Additionally, it was noted that plasma levels of FGF21 remained unaltered in response to short-term (4 h) exposure, and it was speculated that cold-induced BAT FGF21 may benefit itself in an autocrine manner [313]. This assumption was not too speculative, as Hondares et al. had previously demonstrated that injecting fasting newborn mice with FGF21 activated BAT thermogenesis and raised body temperature. This was evidenced by the upregulation of several key thermogenic and oxidative BAT markers like UCP1, PGC-1α, and cytochrome c following injection [314]. On the other hand, Hondares and colleagues later found that rodents enduring longer periods of cold exposure exhibited increased plasma levels of FGF21, leading the authors to consider BAT as a major source of systemic FGF21 upon thermogenic activation [315]. However, some studies have contested the notion that stimulated BAT significantly contributes to the systemic pool of FGF21. Instead, using tissue-specific FGF21 KO models, Ameka et al. and Abu-Odeh et al. found that levels of circulating FGF21 during sympathetic stimulation are primarily determined by the liver’s production despite an observed increase in expression in adipose tissues [316,317]. Regardless, FGF21 is a notable hormonal regulator that has been associated with several metabolic processes, such as lipid metabolism, glucose handling, WAT browning, and, in particular, BAT activity in both mice and humans [318,319,320]. In a randomized controlled trial, Lee and colleagues successfully translated some of the previously mentioned preclinical findings [313,315] into a human context. These authors found that in healthy adults, plasma FGF21 follows a diurnal rhythm and mild-cold exposure augments its circulation levels, which affected the predicted degree of cold-induced thermogenesis (CIT) and lipolysis [321]. In following studies, two independent groups exploring the direct relationship between human BAT activity and circulating plasma levels of FGF21 demonstrated that levels were indeed correlated with BAT activity during cold exposure [322,323]. However, it was more recently observed that cold-induced levels of FGF21 were robustly associated with BAT volume but not necessarily with its thermogenic activity [324]. These observations may be partially explained by Moure et al.’s findings that the thermogenic response of adipose tissue depends on β-klotho levels, a required co-receptor of FGF21 actions. Suppression of β-klotho was shown to alter BAT’s response to chronic cold exposure, lowering normally surged protein levels of UCP1, despite increases in hepatic FGF21 circulation [325]. Conversely, adipose-specific overexpression of β-klotho was shown to increase endogenous FGF21 sensitivity as well as protect mice from DIO. The attenuation of DIO-related metabolic dysfunction was correlated with the induction of several thermogenic genes, namely *Pgc1a*, type II iodothyronine deiodinase (*Dio2*), Solute carrier family 2, facilitated glucose transporter member 4 (*Slc2a4*), and *Ucp1* [326].

#### 5.2.2. BMP(s)

Several members of the transforming growth factor β (TGF-β) superfamily, including bone morphogenetic proteins (BMPs), have been implicated across various stages of adipose tissue development. For instance, BMP 2 and 4 are recognized for their role in mesenchymal stem cell commitment towards the adipogenic lineage [327,328]. Notably, other BMPs have been further reported to influence phenotypic fate. As such, BMP 7 is shown to promote preadipocyte commitment towards the brown fat lineage [329]. Uniquely, in comparison to other BMPs, BMP 7 markedly induced the brown hallmark marker, UCP1, as well as other brown fat-specific genes [329]. Although more controversial, some studies have suggested BMP 4 imparts similar effects. For example, Xue et al. pretreated pluripotent C3H10T1/2 cells with BMP 4 or 7 and found that both activated a full brown-adipogenic program, inducing or upregulating gene expressions related to early brown-fat development, thermogenesis, and mitochondrial biogenesis [330]. Elsen and colleagues similarly demonstrated that the independent exposure of primary human adipose stem cells to either BMP leads to the induction of *Ucp1* expression and reduction in the white-specific marker, transcription factor 21 (TCF21) in primary human adipose stem cells (hASCs) from subcutaneous adipose tissue [331]. Moreover, it was also demonstrated that BMP 4, but not BMP 7, is secreted from fully differentiated human adipose stem cells [331]. Accordingly, it had been previously suggested that secreted BMP 4 from mature adipose may act in a paracrine manner to promote the adipogenic commitment of precursor cells [332]. A possible explanation for the lack of BMP 7 secretion observed in the previous study could be the requirement of a specific stimulus. Boon and colleagues proposed that positive effects associated with BMP 7, such as BAT activation or recruitment, may be contingent upon sympathetic activation. These authors found that mice treated with BMP 7 and housed at 21 °C exhibited an increased total energy expenditure, BAT-specific gene profile, and BAT mass. However, these effects were blunted in treated mice kept at thermoneutrality, 28 °C [333]. BMP8B, a BMP predominantly expressed in BAT [334], was also found to be responsive to thermogenic stimuli and to directly regulate thermogenesis by sensitizing BAT’s peripheral response to adrenergic input [335]. Pellegrinelli et al. corroborated previous observations by demonstrating BMP8B’s involvement in adrenergic-induced neurovascular network remodeling [336]. Before interest in BAT’s potential for combating obesity and its related disorders emerged, it is important to recall that one of BAT’s more fundamental roles was understood to be thermoregulation, specifically in newborns (discussed in the following sections). Therefore, unsurprisingly, in a recent study examining the functional link between human newborn temperature regulation and BAT thermogenesis, it was determined that BMP8B may be involved in neonatal brown-fat adaptation to cold exposure and their overall thermoregulation [337].

#### 5.2.3. Negative Regulators

While most batokines are associated with promoting BAT development or activity, others have been demonstrated to have opposing effects. For instance, the cleaved, secreted form of the LDL receptor relative, LR11, was shown to inhibit thermogenic pathways by disrupting BMP/suppressor of mothers against decapentaplegic homolog (SMAD) signaling. It was suggested that this mechanism is purposeful for preventing excessive energy expenditure during increased thermogenic activity [338]. Endocannabinoids, endogenous lipid-based neurotransmitters [339], have been found to act as autocrine factors that negatively regulate β3-adrenoceptor (β3-AR) -induced BAT activation by suppressing required levels of cAMP [340]. Conversely, Boon et al. demonstrated that blocking the cannabinoid 1 receptor, endocannabinoids’ main receptor that is highly expressed in BAT, induces brown-fat activation, potentially by enhancing cAMP/PKA signaling [341]. Similarly, obstructing the activin receptor IIB, a known integrator of myostatin and other TGFβ-related ligand signaling, was also shown to activate brown adipocyte differentiation and thermogenesis through the inhibition of Smad3 signaling [342]. Notably, myostatin potently downregulates the expression of several of brown adipocyte-related genes, such as PR domain containing 16 (*Prdm16*), *Ucp1*, and *Pgc1a*. These effects were due to myostatin’s ability to induce Smad3 phosphorylation and further the stabilization of β-catenin, a known inhibitor of adipogenesis [343]. Of note, the activity of β-catenin is initially mediated by secreted glycoproteins, collectively referred to as Wnts. Wnt ligands bind to Frizzled receptors, leading to the stabilization and release of β-catenin from the destruction complex. This release allows β-catenin to further modulate adipogenic gene expression [344]. For example, Wnt10b was shown to inhibit brown-fat development by suppressing the induction of the two master adipogenic regulators, PPARγ and CCAAT/enhancer-binding protein (C/EBP) α. However, in the same study, while forced expression of PPARγ and C/EBPα rescued adipogenesis, it failed to restore the cells’ thermogenic capacity (e.g., UCP1 expression). Instead, it was determined that Wnt10b mediates its inhibitory effects on UCP1 expression by suppressing PGC-1α. Therefore, co-expression of PGC-1α and PPARγ was required to negate the inhibitory effects of Wnt10b on both brown adipogenesis and thermogenesis [345]. Despite the fact that our understanding of BAT’s secretome is still evolving, there has been undeniable progress over the last few decades. Given BAT’s crucial role in energy expenditure and metabolic regulation, the bioactive molecules it secretes could be pivotal for obesity intervention, as they have been demonstrated to influence BAT development and thermogenic maintenance. Consequently, batokines stand out as a compelling target for further investigation.

### 5.3. Energy Metabolism

#### 5.3.1. Cold-Induced Thermogenesis

It is widely acknowledged that brown adipose tissue differs from its white counterpart both morphologically and metabolically. As a result, BAT and WAT are known to distinctly contribute to whole-body energy homeostasis. Similar to thermoregulation, maintaining energy homeostasis within a narrow range is crucial as energy imbalances can lead to metabolic disturbances, such as obesity in overnutrition or, conversely, lipodystrophy in undernutrition. As discussed earlier, BAT is a thermogenic tissue that executes its functioning by means of uncoupling oxidative phosphorylation to produce heat and burn energy [298]. This characterized function, in combination with brown fat’s (re)discovery in adults, renewed interest in BAT as a regulator of general energy metabolism and potential therapeutic target for the global epidemic [346]. Over the decades, research has primarily used two methods to stimulate BAT activity: CIT and diet-induced thermogenesis (DIT) [347]. Although rodent models, such as mice and rats, have been the cornerstone of CIT mechanistic studies [348,349,350], human CIT has been proposed to function in a similar manner. This is a reasonable assumption as the framework of CIT encompasses three major factors: (1) response to sympathetic nerve activity, (2) oxidative metabolism, and (3) thermogenic functioning, all of which have been demonstrated in humans [351]. For instance, a strong positive correlation was observed between norepinephrine plasma concentrations and energy expenditure in response to mild-cold exposure or overfeeding in young healthy adults [352]. Fluorodeoxyglucose–positron emission tomography/X-ray computed tomography (FDG-PET/CT) analyses have traditionally served as the method of choice when studying BAT activation due to its non-invasive nature and quantitative reflection of a tissue’s metabolic behavior. Studies using FDG-PET/CT have consistently demonstrated that human BAT is activated in response to cold stimuli [353,354]. As such, van Marken Lichtenbelt et al. observed that 23 of the 24 cold-induced participants in their study exhibited a definite, yet variable, 18F-FDG uptake response in areas where brown adipocytes were confirmed to reside, in particular, the supraclavicular region [355]. A separate study corroborated the previous findings but also identified that FDG uptake in these areas was inversely related to BMI and visceral adiposity [355,356]. Similarly, 4(R,S)-18F-fluoro-6-thia-heptadecanoic acid (18F-FTHA) combined with PET/CT was used to evaluate FA uptake, another vital fuel source of BAT metabolism. It was found that cold stress upregulated both BAT FAU and blood perfusion in lean individuals in a non-shivering manner; however, these effects were diminished in individuals with obesity [357]. Although these studies demonstrated that human BAT responds to cold by increasing its fuel consumption, direct measures of oxidative metabolism were not always included. Therefore, studies have adjusted aspects of their methodologies, particularly in the selection of radiotracers, to more directly address this concern. For example, in addition to using 18FDG and 18F-FTHA, Ouellet et al. opted for 11C-acetate to assess the oxidative capacity of their participants’ BAT. During acute cold exposure, the clearance of ^11^C-acetate in activated BAT, but not other tissues, was significantly increased, indicating enhanced oxidative metabolism [358]. Similar findings have shown that short-term cold acclimation enhances BAT’s oxidative capacity sufficiently to influence the contribution of non-shivering thermogenesis in skeletal muscle [359,360]. Though oxidative activity provides invaluable insight into overall BAT metabolism, further validation of BAT’s thermogenic activity remains pertinent. Therefore, UCP1 expression, the key thermogenic marker, is commonly used to identify functional BAT in humans [312,361]. Again, using FDG-PET/CT as a ‘map,’ tissue biopsies were collected from areas where cold-exposed patients exhibited high glucose consumption and then were further evaluated for classical BAT thermogenic indicators. Indeed, Virtanen et al. found that higher mRNA expressions of the crucial thermogenic marker and several other BAT-specific signatures in BAT biopsies compared to their adjacent WAT equivalents [149]. Moreover, morphological data helped confirm the biochemical findings. While UCP1 expression is a valid marker of thermogenic potential, it does not guarantee its functional activity [362], nor metabolic improvement [363]. Therefore, mitochondrial bioenergetics profiling has been suggested as a more accurate assessment of UCP1-dependent thermogenesis [364]. Porter et al. used high-resolution respirometry on freshly permeabilized brown and white adipose samples from human biopsies to determine mitochondrial respiratory rates [365]. Their results showed that brown adipose has a 50-fold greater respiratory capacity than white adipose, and human supraclavicular BAT responds to guanosine diphosphate similarly to mouse inguinal BAT, suggesting a comparable UCP1 function between human and mice [365]. In a more recent study, cold-stimulated human BAT activation was associated with changes in serum metabolites involved in nicotinamide adenine dinucleotide (NAD)+ metabolism. Of these alterations, the authors reported that cold-induced activation of BAT increased tissue conversion of tryptophan to nicotinamide, a precursor of the NAD+ salvage pathway and indicator of proper mitochondrial functioning. Notably, tryptophan levels are inversely related to UCP1 expression, thus indicating its cold-activated utilization [366].

#### 5.3.2. Diet-Induced Thermogenesis

Despite the fact that the cold is a potent and reliable stimulus for brown-fat activation, its impracticality limits its benefits for humans. Consequently, research has been investigating the thermogenic potential of DIT, given its contribution to daily energy expenditure [367,368]. Furthermore, it has been observed that increases in BAT oxygen consumption in humans following a carbohydrate-rich meal are comparable to those observed during CIT [369]. DIT refers to the body’s physiological response to caloric intake by which energy expenditure exceeds basal metabolic rate during food processing [368]. The energetic response of DIT can be further subdivided into its obligatory and facultative components, with the latter emphasizing BAT’s assumed contribution to energy metabolism [370]. Indeed, rodent models have demonstrated that calorie intake activates BAT, increasing thermogenic parameters, such as respiration rate, glucose utilization, fatty acid synthesis, and mitochondrial guanosine diphosphate binding [371,372,373]. In addition, rodent studies have identified UCP1 as an essential component in the mediation of diet-induced thermogenesis [374]. For example, Feldmann and colleagues demonstrated that UCP1 ablation not only induces obesity but also abolishes DIT in mice living at thermoneutrality [375]. Another study has corroborated these findings, reporting both the occurrence of obesity and a lack of evidence for DIT in UCP1-deficient mice, despite these mice originating from an obesity-resistant strain [376]. Unfortunately, human evidence regarding BAT DIT is less convincing due to the (1) inconsistencies found in the available, yet (2) limited data. Using whole-room indirect calorimetry, DIT and substrate utilization were measured in participants with and without 18F-FDG-PET/CT-detected BAT activity. The study found that BAT-positive participants had a higher energy expenditure and relative DIT (%), along with a lower respiratory quotient after meals, suggesting that DIT may contribute to energy metabolism in healthy subjects with metabolically detected BAT [377]. However, in a similar study, Loeliger et al. did not find an association between glucose-stimulated BAT and DIT and concluded that DIT is likely not an imperative function of human BAT [378]. Furthermore, a recent meta-analysis determined that diet and nutrition do not significantly affect human BAT activity [379]. Although human studies commonly use indirect calorimetry methods to support theories regarding BAT DIT, the inability of this approach to properly differentiate between various energy types presents a significant drawback, as it tends to overestimate the contribution of heat energy dissipation during the postprandial state. This issue was thoughtfully addressed in the review article “Diet-Induced Thermogenesis: Fake Friend or Foe?” by Ken K. Y. Ho, where Ho advises of the need to complement indirect calorimetry with other methods, such as direct calorimetry or infrared thermography, to improve the accuracy of evidence in future research [380]. Moreover, even at the preclinical level, the late L. P. Kozak had voiced early skepticism regarding the contribution of BAT DIT to energy homeostasis [381]. The widely accepted underlying premise of BAT DIT, like CIT, revolves around the functioning of UCP1. However, works from his lab challenged the legitimacy of this view, revealing intriguing evidence of resistance to hyperphagia and diet-induced obesity in their *Ucp1* knockout mice [382,383,384,385]. To-date, findings from DIT studies are still fairly inconsistent, and these discrepancies have been attributed to various factors, such as small sample size or methodology [386]. However, NST mechanisms, both UCP1-dependent and independent, outside the scope of BAT exist and are being considered as an option to counter obesity. One of these approaches is the metabolic remodeling of the ‘culprit’ itself, WAT [387,388,389].

## 6. Browning of WAT

### 6.1. Stimuli

Before the phenomena of “browning” became widely acknowledged, Young et al. reported the peculiar detection of brown adipose within a fat pad commonly known to be exclusively white. In addition, they also noted that the density of this brown tissue increased when mice were exposed to cold temperatures [390]. Unknowingly, Young and colleagues had just helped lay the groundwork for the modern concept of browning, where intrinsic white adipocytes respond to stimuli and transform into an intermediate adipose type that exhibits characteristics of both white and brown types. Nearly five decades later, researchers are still investigating the molecular and functional dynamics of WAT browning, with many supporting its potential to combat obesity and related comorbidities [391].

#### 6.1.1. Cold and Pharmacological Agonist

Cold exposure is frequently used in BAT research to promote its thermogenic function and is also a potent inducer of WAT browning [392]. This activation relies on sympathetic nervous system signaling for both BAT activation and WAT browning [393,394]. For example, in adult Sv129 female mice, cold acclimation increases noradrenergic branching and the number of *Ucp1*-positive adipocytes within white-fat depots [395]. Additionally, β3-adrenergic receptor activation, a primary receptor mediating the cold-induced effects in BAT, is essential for the transdifferentiation of white adipocytes into brown-like cells [396]. Supporting this, systemic treatment with a selective β3-AR agonist increases UCP1 expression and promotes BAT-like characteristics in inguinal WAT [397]. Radiotracer experiments further show that β3-AR agonists can enhance the metabolic activity of both interscapular BAT and inguinal WAT [398,399]. However, in cold-exposed mice lacking β3-AR signaling, the transdifferentiation of white to brown adipocytes is significantly diminished [400]. The most clinically relevant β3-AR agonist being used to elucidate the potential of sympathetic nervous system (SNS)-mediated WAT browning is the FDA-approved drug mirabegron. While initially developed for treating overactive bladders [401], clinical trials have demonstrated that mirabegron also exerts several metabolic effects, such as improving glucose tolerance and insulin sensitivity. Notably, these effects are associated with subcutaneous WAT browning (a.k.a. “beigeing”) [402,403,404]. More recent preclinical work has suggested that the browning of adipose tissue induced by mirabegron may be linked to the drug’s anticancer activity. It was proposed that mirabegron-induced tumor suppression is instigated by a UCP1-facilitated shift in glucose metabolism [405]. Remarkably, a post-authorization safety study observing over 5000 cancer incident reports found no association between mirabegron use and overall cancer risk [406]. However, in a subgroup analysis, it was determined that higher cumulative doses of mirabegron significantly increased kidney cancer incidence in comparison to lower doses. In addition to perinephric fat browning, the authors also reported that β3-AR agonists modulate tumor immune tolerance, which promotes cancer initiation and progression [407]. Other researchers caution that mirabegron-induced browning of WAT and/or activation of BAT may also elevate cardiovascular and cerebrovascular risks by exacerbating atherosclerotic plaque development [408]. However, opposite data obtained by Ying and colleagues determined that mirabegron not only induces WAT browning and stimulates BAT activity but also improves lipoprotein profiles that are associated with reduced atherosclerotic lesion size. The authors suggest that the discrepancies found between studies may be due to the specific characteristics of [408] selected model, which lacks hepatic TRL remnant clearance—an aspect not representative of the general human population [409]. Despite the conflicting results, clinical evidence suggests that a 100 mg dose of mirabegron can promote beneficial metabolic effects, such as increased energy expenditure, without elevating cardiovascular risks [410]. Supporting this, another group of investigators found that mirabegron’s effects are comparable to cold exposure, as both increased lipid oxidation, FFAs, and skin temperature while decreasing the brown-fat fraction [411]. A recent meta-analysis corroborated previous findings by concluding that mirabegron increases various parameters, including BAT activity, body temperature, resting energy expenditure, and non-esterified fatty acids. However, it was shown that mirabegron also modulates heart rate, blood pressure, and blood insulin levels, suggesting it may be a viable option for managing obesity-related comorbidities, such as cardiovascular disease and diabetes [412]. Despite the overall clinical potential of mirabegron and the reliability of cold exposure to promote the browning of white fat and/or modulate BAT activity, both mechanisms have limitations, such as off-target effects [413] or impracticality, respectively. This highlights the importance of more sustainable lifestyle approaches, like diet and exercise, in promoting adipose tissue browning and improving metabolic health.

#### 6.1.2. Exercise

Exercise is known to benefit human health in many ways, some of which include improved sleep and cognitive function, mood stabilization, immune support, and prevention of certain diseases [414]. In addition, exercise has also been demonstrated as a browning agent that integrates factors from various organ systems to promote its effects. For instance, exercise training has been shown to induce the production and release of several exerkines from various tissues that ultimately converge to regulate UCP1 expression in WAT [415]. Among these are the PGC-1α-regulated expression of fibronectin type III domain-containing protein 5 (FNDC5). During exercise, PGC-1α stimulates FNDC5 expression in skeletal muscle, which is subsequently cleaved and then secreted in the form of irisin. Circulating irisin can then act on WAT to upregulate a brown-fat-like program [416]. This muscle–adipose crosstalk is further supported by Xiong et al.’s loss-of-function study that demonstrated that exercise-induced browning of WAT is diminished in their Fndc5 mutant mice [417]. It has also been suggested that TGF-β/SMAD signaling may play a role in WAT browning by suppressing the expression of FNDC5 and PGC-1α in skeletal muscle, thus reducing the production and/or secretion of irisin [418]. Further downstream of irisin’s initial cascade, CD81, a brown-fat-like progenitor surface protein, was shown to mediate integrin-focal adhesion kinase signaling in order to regulate the proliferation and biogenesis of brown-like progenitor cells [419]. While preclinical trials of irisin support its potential to promote browning and benefit metabolism, human studies are less consistent. In 2012, Boström et al. reported a significant increase in *FNDC5* mRNA from muscle biopsies, with circulating irisin levels doubling in subjects after a controlled endurance training period. The authors suggest that irisin may play a role in mediating the metabolic benefits of exercise, potentially protecting against several human metabolic disorders [416]. Conversely, a study conducted one year later by Pekkala et al. reported opposite findings. They found no significant changes in either *FNDC5* or serum irisin levels after exercise, regardless of training type—whether aerobic, endurance, or a combination of endurance and resistance. The authors suggest that other factors may regulate *FNDC5* expression and irisin release, as their data showed an inconsistent correlation between *PGC-1A* mRNA expression and changes in *FNDC5*. Additionally, they further noted that neither FNDC5 nor irisin was associated with metabolic parameters, such as glucose tolerance, insulin sensitivity, and/or obesity status [420]. Others have corroborated these findings, with some studies reporting that the expression of *FNDC5* or its product irisin does not even correlate with *UCP1* mRNA in WAT [421,422,423]. These translational discrepancies urge researchers to invest time and resources elsewhere by either exploring the browning potential of other exerkines, such as FGF21 [424,425], interleukin 6 [426,427], growth differentiation factor 15 [428,429] and/or alternative approaches, including dietary components.

#### 6.1.3. Nutraceuticals

Research suggests that certain natural compounds from various food sources may promote the conversion of WAT to a more thermogenically active and brown-like state, a process linked to increased energy expenditure and potential anti-obesity benefits.

Resveratrol—a polyphenol naturally found in the skins of berries and grapes—has been shown to encourage WAT browning [430] by modulating the activity of sirtuin (SIRT) 1, a deacetylase enzyme. Studies indicate that both resveratrol and resveratrol-like compounds regulate SIRT1 activity [431,432], leading to the deacetylation of PPARγ at specific lysine residues: Lys268 and Lys293. This deacetylation facilitates the recruitment of the thermogenic coactivator PRDM16 to PPARγ. As a result, brown-fat-specific genes, such as *Ucp1* and *Dio2*, were shown to be upregulated while white-associated genes were repressed [433,434]. In addition, others have suggested that resveratrol may also induce brown-like adipocyte formation and enhance thermogenic function by activating the cellular energy sensor AMP-activated protein kinase (AMPK) α1 [435,436]. Interestingly, resveratrol has also been associated with regulating several of the aforementioned exerkines, including both FNDC5/irisin [437] and FGF21 [438].

Capsaicin—a compound extracted from chili peppers—is another dietary component investigated for its potential to induce WAT browning and promote anti-obesity effects [439]. Research by Baskaran and colleagues demonstrated that capsaicin promotes the expression of brown-fat-specific genes, including *Ucp1* and *Bmp8*, in WAT. Capsaicin has also been shown to increase SIRT1 expression and activity, enhancing the interaction between PPARγ and PRDM16 similarly to resveratrol. This capsaicin-induced SIRT1 activation is mediated through a TRPV1 channel-dependent influx of intracellular calcium and the phosphorylation of AMPK and Ca^2+^/calmodulin-activated protein kinase [440]. In addition to capsaicin, other TRPV1-activating compounds have been found to promote WAT browning. Zhang et al. reported that hydroxy-α-sanshool, an active amide from the fruits of Zanthoxylum bungeanum Maxim, can activate the TRPV1/AMPK pathway, leading to PPARγ deacetylation and WAT browning [441]. Hydroxy-α-sanshool may serve as an alternative for individuals sensitive to the spiciness of capsaicin, as it provides a similar thermogenic effect without the same level of pungency. This should be considered, as some research suggests capsaicin, unlike its non-pungent analogs, is necessary for maximal TRPV1 activation and thermogenic response in WAT [442].

Another dietary component shown to induce WAT browning is fish oil [443]. For example, Kim and colleagues conducted a study demonstrating that mice fed fish oil (FO) began to exhibit a gene expression profile in their inguinal WAT that closely resembled that of BAT. This included key BAT-specific and thermogenic markers, such as UCP1 at the protein and gene levels, and *Pgc1a*, *Prdm16*, carnitine palmitoyltransferase 1b (*Cpt1b*), and cell death-inducing DFFA-like effector a (*Cidea*) at the gene level. In addition, the authors noted that both oxygen consumption and body rectal temperature were increased in response to FO feeding. These effects were concomitant with the upregulation of *Ucp1* and the β3-adrenergic receptor (β3AR) gene (*Adrb3*), implying that fish oil may mediate its thermogenic effects via SNS activation [444]. Other researchers have noted similar effects of fish oil, highlighting that mice on a high-fat diet supplemented with fish oil exhibit an increased expression of genes associated with mitochondrial biogenesis and β-oxidation in both epididymal and subcutaneous fat pads [445]. Furthermore, Yamazaki et al. proposed that fish oil could be a beneficial dietary fat associated with DIT. Mice given FO exhibited a significant increase in several BAT/thermogenic markers, such as *Ucp1* and Cidea, as well as other genes involved in β-oxidation, within their subWAT. Notably, the expression of *Adrb3* was also found to be higher in mice fed FO compared to the control-fed group. This led to the speculation that FO might induce a thermogenic gene profile in subWAT via SNS [446]. In short, there are many ways to “brown” WAT, some more well-established than others, and the novelty of each browning agent has been discussed extensively in their own niche reviews [447,448]. However, the outcome of browning, regardless of what agent was used, is always the same: an intermediary adipocyte that resides in white fat but can function similarly to brown fat. Therefore, if the end goal is the same, then why does the literature entertain a catalog of synonyms when referring to the same adipocyte type?

### 6.2. Brown-like Adipose

The fundamental framework of WAT browning is solid. Converting excess white adipose tissue, as seen in obesity, into a type that might eventually resolve itself is a promising approach. However, the field seems to lack agreement on how to term these brown-like adipocytes, with some referring to them as ‘beige’ and others as ‘brite’. Moreover, just to add to this perplexity, the literature further accommodates a range of acronyms (‘BeAT’) [449,450], adjectives (‘inducible,’/‘recruitable,’/‘convertible’) [152,153,451,452,453], and novel pseudonyms (‘taupe’) [454]. Conventionally, many authors attempt to resolve this conundrum by lumping terms together when discussing their results [152,455]. However, returning to the start of this Section 6.1 may prompt a reconsideration of this grouping method. Young et al.’s report highlights several key findings: (1) the detection of brown “areas” within an otherwise known white-fat pad at a standard mouse housing temperature (~23 °C) and (2) the intensification and propagation of these brown areas upon cold exposure [390]. Therefore, Young et al.’s study was not only pioneering in documenting the browning phenomenon, but their results also suggest the presence of distinct cell populations that contribute to this process.

#### 6.2.1. Markers of Identification

In order to fully elucidate the potential of beige adipocytes and their contribution to human health and obesity, it is first necessary to properly identify this subcategory of cells. Although this objective is rather challenging, as these adipocytes are known to be the intermediate of both WAT and BAT, several research groups have proposed a variety of enriched transcripts that may be unique to this population [450,456]. For instance, Wu et al. identified a pool of progenitors from the SVF of the inguinal depot that were reminiscent of, but not absolutely identical to, classical brown adipocytes. After differentiation, these inguinal-derived cells expressed basally low levels of several brown-fat genes, including *Ucp1*, cytochrome c oxidase subunit 7 isoform A1 (*Cox7a1*) and *Cidea*. Importantly, basal levels of these genes from this pool were comparable to the unstimulated levels in the subset determined as white. However, upon cAMP stimulation, this pool of adipocytes, but not the white subset, exhibited a large induction of *Ucp1* and uncoupled respiration that either mirrored or outperformed brown adipocytes’ response, respectively. Transplantation experiments confirmed these cells’ ability to respond to SNS input and that the enhanced UCP1 gene expression observed was reproducible in an in vivo setting [457]. Differential gene analysis revealed several unique signatures of these responsive, inguinal-derived cells, such as transmembrane protein 26 (*Tmem26*), cluster of differentiation (CD) 137 (*Cd137*), and T-box 1 (*Tbx1*). Furthermore, fluorescence-activated cell sorting of primary inguinal SVF residents expressing either *Cd137* or *Tmem26* corresponded with groups of cells that exhibited basally elevated *Ucp1* expression [457]. Additionally, recognized beige-selective markers, such as Cbp/p300-interacting trans-activator with Glu/Asp-rich carboxy-terminal domain 1 (CITED1) and SHOX homeobox 2 (*SHOX2*), have been detected in human BAT [458,459]. Beige-selected adipocytes using the above criteria were found to be *UCP1* (+) and correlated with the brown-fat-selective gene *PRDM16* [458,459,460]. It has been proposed that PPAR agonists, such as rosiglitazone, can function synergically with PRDM16 to confer the brown fat gene program in subcutaneous WAT [461]. However, other lines of evidence have suggested that the brown-fat-selective marker does not maintain a role in regulating the beige-induced phenotype in white depots and, instead, is only prioritized in the muscle relating to brown-fat fate [460,462]. In 2015, several of the proposed beige-selective markers, such as *Cd137*, epithelial stromal interaction 1 (Epsti1), Tbx1, Tmem26, carbonic anhydrase 4 (*Ca4*), *Cited1*, *Fgf21*, and homeobox C9 (*Hoxc9*), underwent an assessment where their validity as beige-fat markers was critically revisited. De Jong et al. concluded that, among the growing number of proposed beige markers, *Cd137*—and to an even lesser extent, *Tbx1* and *Tmem26*—show the most potential as reliable marker genes at the tissue level. However, none of these markers were exclusively expressed in primary inguinal WAT cultures, highlighting the need for deeper assessment of the currently proposed markers and the potential identification of novel ones [463]. Accordingly, single-cell RNA sequencing recently enabled the identification of an adipose stem cell subpopulation within the inguinal WAT depot that exhibits the propensity for a beige phenotype in response to cold stimuli. Nahmgoong et al. characterized this subpopulation by the cell surface marker bone marrow stromal antigen 2^high^ and, upon differentiation, found that these inguinal-derived adipose stem cells begin to highly express thermogenic markers, including *Ucp1* and *Dio2*. In addition, it was noted that these cells were derived from a cluster also associated with beige expressions, including *Tmem26* and *IL33*. Moreover, cold exposure was shown to increase the proportion of these bone marrow stromal antigen 2^high^ ASCs, whereas high-fat feeding was shown to downregulate them and, correspondingly, *Ucp1* expression [464]. Without a doubt, more sophisticated methods, such as single-cell RNA sequencing and large-scale bulk analyses, have significantly advanced our understanding and precision in distinguishing beige/brite adipocytes from classical WAT and BAT. However, the pesky challenge of pinpointing how these cells emerge still baffles researchers today—is it by de novo beige adipogenesis? WAT to brown-like transdifferentiation? or both?

#### 6.2.2. Origins

It is widely accepted that these cells are indeed their own entity, not too brown and not too white, but just ‘brite.’ Evidence collected throughout the history of browning research has led studies to support each theory. While some investigators are convinced this subset of adipocytes arises from a distinct precursor, others contemplate the possibility of mature transdifferentiation. For example, after rats were treated with the β3AR agonist, CL-316243, ~17% of their retroperitoneal WAT adipocytes exhibited a multilocular appearance, compared to only about 0.3% from the control group. Furthermore, treated cells were almost completely negative for BrdU, suggesting that the accumulation of multilocular cells was not due to mitotic proliferation of precursors but instead the conversion of pre-existing unilocular cells [453]. Nearly a decade later, Barbatelli et al. supported the previous findings by demonstrating the occurrence of WAT-derived adipocytes that were UCP1-immunoreactive and exhibited a mixed morphology (paucilocular) following cold exposure. Importantly, quantitative electron microscopy determined these cold-induced paucilocular cells were not due to an induction of cell proliferation but again, by transdifferentiation [396]. Conversely, other reports from the same era, such as the study by Petrovic and colleagues, challenge this view and argue that the UCP1-induced adipocytes within the ‘purest’ WAT depots are in fact a molecularly distinct subcellular population. The authors conducted co-culture experiments to eliminate the possibility that a few preexisting brown preadipocytes might have simply overgrown and outcompeted white adipocytes during stimulation. Despite PPARγ activation causing a subset of epididymal-derived preadipocytes to acquire thermogenic capacity, rosiglitazone-treated cultures lacked many origin-specific myogenic and brown fat markers. Additionally, they seemed to exhibit markers of white fat origins, such as homeobox C9, distinguishing them from both classical brown and white adipocytes [462]. Vegiopoulos et al. demonstrated that cyclooxygenase 2—prostaglandin signaling lies downstream of the canonical β-adrenergic pathway and may play a role in shifting the differentiation of WAT mesenchymal progenitors towards a brown-fat phenotype. Importantly, and consistent with Petrovic et al.’s findings, classic brown-fat transcripts, including *Myf5* and LIM/homeobox protein Lhx8 (*Lhx8*) genes, were not found to be enriched in either the CL-316243-induced or cyclooxygenase 2-overexpressing brown-like adipocytes, indicating their idiosyncratic origin [465]. Human studies have also indicated a separately defined and inducible subpopulation of adipocytes that reside within either known classical brown (cervical and supraclavicular) or white (periadrenal) depots [452,466,467,468]. Taken together, emerging evidence supports both perspectives, but future research must address key considerations to optimize the clinical translation of adipose browning. Critical questions include defining the precise molecular signatures of distinct cell populations and identifying targetable genes to control their differentiation fate. Does stimulus type matter (e.g., cold exposure versus dietary versus pharmacological interventions), and if so, what thresholds are required for functional thermogenesis that is clinically meaningful? Can the chosen pharmacological agents achieve physiologically relevant doses in humans, or are the stimuli even feasible? Beyond these hurdles, what are the turnover rates, sustainability, and long-term clinical implications of browning once stimuli are withdrawn? Would it prove more effective to expand novel progenitor cells ex vivo or reprogram mature white adipocytes in situ? Finally, do transdifferentiated cells revert to a functional white adipocyte state, or do they undergo stable epigenetic reprogramming that alters their response to future stimuli? Resolving these issues will be essential before positioning adipose browning as a viable anti-obesity therapeutic strategy.

## 7. Yellow Adipose Tissue (YAT)

**Figure 2:**
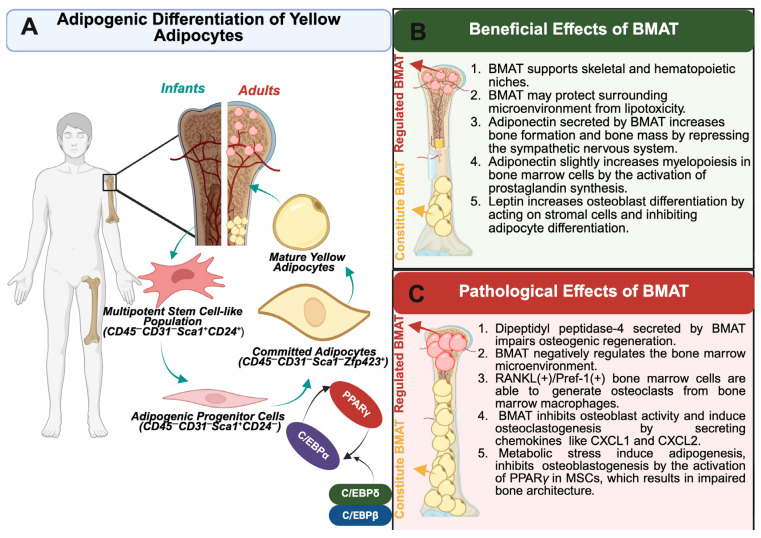
Relevance of yellow adipose tissue. YAT, also referred to as BMAT or MAT, is an emerging adipose tissue type under investigation due to its proposed impact on human health and disease [472,473]. In panel (**A**), we briefly summarize the maturation of premature yellow adipose from multipotent stem cells (MSCs) to fully differentiated yellow adipocytes. In panels (**B**,**C**), both the beneficial [469,474,475,476,477,478,479,480] and pathological [481,482,483,484,485,486] effects of YAT are outlined. Created in BioRender. Dowker, P. (2026) https://BioRender.com/f40i747 (Accessed on 10 February 2026). Footnotes & abbreviations: Bone marrow adipose tissue (BMAT), CCAAT/enhancer-binding protein beta (C/EBPβ), CCAAT/enhancer-binding protein delta (C/EBPδ), cluster of differentiation 24 (CD24), cluster of differentiation 31 (CD31; platelet endothelial cell adhesion molecule-1 or PECAM-1), cluster of differentiation 45 (CD45; leukocyte common antigen), C-X-C motif chemokine ligand 1 (CXCL1; keratinocyte chemoattractant in mice), C-X-C motif chemokine ligand 2 (CXCL2; macrophage inflammatory protein-2 or MIP-2 in mice), mesenchymal stem cell (MSC), peroxisome proliferator-activated receptor gamma (PPARγ), preadipocyte factor 1 (PREF-1; also known as DLK1), receptor activator of nuclear factor kappa-B ligand (RANKL), stem cell antigen 1 (SCA1; Ly6A/E in mice), zinc finger protein 423 (Zfp423).

While most preclinical research has traditionally focused on modulating white and brown adipogenesis, yellow adipose tissue, a fat depot localized within the bone marrow, has received little attention despite a growing body of evidence implicating it in human health and disease. Notably, YAT [a.k.a. bone marrow adipose tissue (BMAT) or marrow adipose tissue (MAT)] depots have been observed within the medullary canal of long bones (tibia, femur, and humerus), ribs, vertebrae, and sternum [469] and make up 10–15% of total adipose mass [470,471] (Figure 2).

Although still controversial, YAT has been initially proposed to originate from various MYF5-negative progenitor stem cells under the control of pRb [487,488,489]. However, recent studies argued that yellow adipocytes originate mostly from skeletal lineages [471,490,491]. A recent study by Ambrosi and colleagues delineated the molecular identity of the bone-resident yellow adipocytes and determined that only progenitor cells that are negative for both hematopoietic *Cd45* and endothelial *Cd31* markers but positive for mesenchymal *Sca1* marker give rise to bona fide mature adipocytes within the bone marrow [481]. Furthermore, Scheller and colleagues identified two functionally different marrow adipose tissues: proximal ‘regulated’ MAT (rMAT) and distal ‘constitutive’ MAT (cMAT), fats with distinct roles in hematopoiesis. rMAT appears in the form of scattered single adipocytes with active hematopoiesis, whereas distal cMAT contains larger adipocytes and displays low hematopoiesis [469]. Taken together, these studies point to a unique type of fat with potentially critical functions in health and disease.

Functional YAT depots exhibit several unique features; however, under certain conditions, characteristics that resemble either BAT or WAT may emerge. For instance, despite their histological resemblance to white adipocytes with unilocular lipid droplets, marrow adipocytes express several markers of beige and brown adipocytes, including *Tbx1*, *Dio2*, *Ucp1*, *Prdm16*, *Pgc1a* [492]. As such, YAT depots have been shown to not only regulate hematopoiesis and bone remodeling, but they also play a critical role in energy balance and systemic metabolic homeostasis through both endocrine and paracrine functions [471]. Cawthorn and colleagues found that caloric restriction increases bone marrow adipose tissue mass concomitant with an elevation in the levels of circulating adiponectin [493]. Interestingly, the levels of secreted adiponectin were higher in MAT compared to WAT and were also increased in patients receiving cancer therapy [493] and mice treated with rosiglitazone [494]. Given the role of adiponectin in several biological and physiological processes in addition to metabolic homeostasis, such as neurogenesis, cardiovascular function, the immune system, and a plethora of other systems [493], it is reasonably arguable to assume that alterations to YAT development and function may contribute to the pathogenesis of several metabolic and non-metabolic diseases. As such, a deep understanding of the biology of this tissue may yield novel insights into its role in health and disease. This is also supported by the fact that YAT may exhibit changes to its metabolic activity in response to various physiological, environmental, and pharmacological stimuli, including aging [481,495,496], cold exposure [497,498], and treatment with Metformin, PPAR agonists, and several other drugs [499,500,501,502].

While early studies have established a strong association between obesity and bone marrow adipose tissue mass and inflammation [503,504], this association appears to be modulated by several factors. For instance, adipose tissue occupies the majority of long-bone marrow cavities and represents only 40% of iliac crest marrow, with the remaining 60% being hematopoietic cells [505]. Additionally, the distribution of bone marrow adipose tissue is also affected by other factors, such as ethnicity and health status, and can also be site- and species-specific, as bone marrow mass and location are different in human versus experimental models of human obesity [157,506]. These differences possibly suggest that the mechanisms of bone marrow adipogenic differentiation may also be affected by these factors and warrant further investigations [506]. In healthy premenopausal women, a positive correlation between bone marrow fat mass and visceral adiposity, independent of bone mineral density, was observed [503]. In line with these observations, exercise-induced body weight loss in mice was associated with a decrease in bone marrow fat mass and improved bone health [507]. Of note, increased bone marrow fat mass has been associated with several detrimental metabolic alterations. For example, Ermetici et al. demonstrated a negative correlation between bone marrow fat mass and insulin sensitivity in obese and non-obese premenopausal women [508], possibly caused by alterations to adiponectin and leptin levels and the subsequent effects on insulin sensitivity and glucose homeostasis [508,509]. According to this study, the increase in bone marrow fat mass could be attributed to a compensatory effect caused by changes to the levels of C/EBPα/δ and PPARγ2, all of which are key players in adipogenesis.

In support of its contribution to metabolic homeostasis, recent attention identified a potential relationship between bone marrow fat mass and diabetes, both type 1 and type 2, and the related skeletal fragility. However, while human studies have yet to provide robust evidence of such an association [510,511,512], experimental rodent models of type 1 diabetes (T1DM) often exhibit higher bone marrow fat mass compared to healthy animals, concomitant with lower bone mass [513,514,515]. Notably, Martin and McCabe demonstrated that bone marrow adiposity in diabetic mice could be location-dependent, as it is higher in the femurs and calvaria area but not in the vertebrae area [515]. In support of this observation, despite their lower peripheral adiposity and lean mass, diabetic C/EBPβ deficient mice displayed a five-fold increase in bone marrow adiposity compared with controls. These effects were concomitant with alterations to bone health as judged by the reduced bone stiffness and enhanced bone resorption in diabetic mice [516]. The increase in bone marrow fat mass was also evident in the streptozotocin-induced T1DM experimental model, where treatment with streptozotocin increased the differentiation of mesenchymal stem cells toward an adipocyte lineage [517]. However, despite the positive association between T1DM and bone adiposity, there is insufficient data to support a causal relationship. While treatment of T1DM with insulin ameliorated bone health, it failed to prevent adipocyte differentiation within the bone marrow, suggesting that other factors in addition to bone marrow fat cells contribute to bone loss in T1DM and require further investigation. On the other hand, treatment of insulin-deficient diabetic BALB/c with bisphenol-A-diglycidyl ether, a PPARγ agonist, blocked bone adiposity but failed to prevent diabetes-induced bone loss [518]. Taken together, these findings, while they establish an interplay between T1DM and bone fat mass, also advocate for a complex relationship that requires further examination.

The reciprocal relationship between bone marrow adiposity and glucose homeostasis has also been observed in T2DM, both in humans and rodent models. HbA1c levels were positively associated with higher marrow adiposity in adult men with morbid obesity [519]. Similarly, significant positive correlations were observed between vertebral bone marrow fat and T2DM in elderly men [520]. Conversely, treatment of healthy postmenopausal women with the antidiabetic drug rosiglitazone reduced bone marrow adiposity [521]. However, while the association between T2DM and bone marrow fat mass in humans has been challenged by other studies [522,523], experimental models of T2DM consistently demonstrated increased bone marrow fat mass in T2DM animals. Early onset of T2DM correlated with up to 50-fold-higher bone marrow adiposity in Tallyho mice [524]. Likewise, an increase in bone marrow fat mass was observed in high-fat-feeding-induced obesity and T2DM [525]. Although no effects on peripheral adiposity were observed upon treatment with metformin, a first-line treatment of T2DM, both bone marrow fat mass and T2DM were reversed [525]. These studies provide evidence for the role of bone marrow fat in the regulation of systemic energy metabolism and glucose homeostasis, a hypothesis that is further corroborated by studies demonstrating the ability of bone marrow fat to respond to insulin-sensitizing agents [499,500,525,526].

In addition to its role in metabolic regulation, several early and recent studies have identified bone marrow fat as a negative regulator of hematopoiesis [485,527,528,529]. Although the exact molecular mechanisms are yet to be determined, Harada and colleagues demonstrated that plasminogen activator inhibitor type-1 (PAI-1) is responsible for the detrimental effects of bone marrow fat on hematopoietic regeneration [528]. PAI-1 is produced by several tissues, including fat, and functions as a serine protease inhibitor. It has also been linked to the pathogenesis of several acute and chronic pathophysiological processes, including cardiovascular disease, tissue fibrosis, cancer, and age-related diseases [530]. PAI-1 not only affects the movement and retention of hematopoietic stem and progenitor cells within the bone marrow [531] but also plays a role in regulating insulin signaling in peripheral tissues, contributing to insulin resistance and metabolic dysfunction [532].

Given its role in systemic inflammation and obesity, it is not surprising that bone marrow fat mass is also associated with bone and metabolism [533]. Indeed, higher bone marrow fat mass has been linked to decreased bone mineral density and increased fracture risk [534,535,536]. Additionally, the lipid composition of bone marrow fat and the relative amounts of saturated versus unsaturated fat were demonstrated to have an impact on skeletal health, bone density, and fracture risks [537,538]. As demonstrated by Yeung and colleagues [539] and further confirmed by Li et al. [540], the fat unsaturation index was significantly lower in osteoporotic and osteopenic postmenopausal women compared to controls. Further research is needed to fully understand the molecular mechanisms mediating the association between bone density and bone marrow fat mass and composition. However, a study by Fazeli and colleagues investigating the relationship between several adipokines, fat mass, and bone mineral density in women with anorexia nervosa found an inverse association between bone mineral density of both the anteroposterior spine and lateral spine and the expression of several adipokines [541]. This includes preadipocyte factor-1, insulin-like growth factor (IGF)-I, IGF-binding protein 2 and leptin; all of which are well-established modulators of adipocyte and osteoblast differentiation, but also bone growth and density. For instance, IGF-I was demonstrated to directly regulate bone growth and density [542]. Likewise, an increase in the levels of IGF-II/IGF binding protein-2 complex stimulates bone formation and prevents loss of bone mineral density in a rat model of disuse osteoporosis [543]. Similarly, systemic administration of leptin in animals and humans was often associated with positive outcomes on bone health [544]. The role of preadipocyte factor-1 in bone health, on the other hand, is controversial and warrants additional investigation [545,546,547,548]. Together, while research highlights adipokines as crucial for bone health, the specific role of bone marrow fat in circulating these adipokines is still unclear. However, the observed link between bone density and bone marrow fat mass/composition suggests involvement of multiple mechanisms, with adipokines being a strong possibility.

## 8. Pink Adipose Tissue (PAT)

Recent advances in the field, led by Saverio Cinti from the University of Ancona, Italy, led to the identification and characterization of a novel type of lipid-rich parenchymal cell within the mouse mammary glands called “pink adipocytes.” These cells develop and are maintained during the maternal stages of pregnancy and lactation; however, they transdifferentiate into white and brown adipocytes upon the post-lactation period [155,549,550]. Pregnancy provokes white, brown, and brown-like adipocytes within the anterior and posterior subcutaneous depots to enter the “mammary gland cycle” to form lobuloalveolar structures (alveoli; alveologenesis) that support a milk-producing/secreting organ. This transdifferentiation occurs in two phases; first, alveoli develop as a result of possibly increased stem cell proliferation during the early stages of pregnancy. Then, intracellular lipid droplets emerge inside these cells during the second stage of pregnancy, leading to the formation of pink adipocytes, which occurs concomitantly with the shrinkage of subcutaneous fat [551] as the mammary ducts substitute most of the subcutaneous adipose tissue [552,553,554,555]. The post-lactation phase ultimately replaces the newly constructed alveoli with adipocytes, thus restoring it back to the original form: a subcutaneous depot infiltrated by branched epithelial ducts [155,551]. Ductal stem cell recruitment, transformation, and subsequent apoptosis form the fundamental essence of the leading hypothesis attempting to explain the adipocyte/epithelial transdifferentiation [549,556,557,558]. Although its clinical relevance is yet to be determined, the transdifferentiation of pink adipocytes into brown or brown-like adipocytes post-lactation [155,559]. insinuates their potential role in the metabolic homeostasis and remodeling of the surrounding microenvironment and warrants additional investigation.

## 9. Conclusions and Future Directions

It is clear that obesity research has come a long way since its declaration as a global health crisis. Importantly, we exist in an era where healthcare is adopting a more interdisciplinary approach that considers multiple expertise in order to provide the most efficient patient care [560]. It is reasonable to speculate that benchtop research may greatly benefit from this model as well. Therefore, in this review, we have explored the intricacies of the body’s different adipose tissues, examining each type’s unique and functional contributions to human health. Identifying areas of overlap and divergence among the various adipose tissues will allow for a honed, yet more comprehensive, perspective when addressing the rising epidemic. However, despite the field’s major advances on the topic, we are continuously seeing novel, and sometimes even revisited, avenues within adipose/obesity research. For instance, in more recent years, glucagon-like-peptide-1 (GLP-1) receptor agonists (RA), such as Liraglutide and Semaglutide, have been in high demand, particularly on account of their FDA approval and success for weight management in both diabetic and non-diabetic patients [561,562]. Various metabolic attributes, besides just weight loss and glycemic control, have been associated with the prescription of these drugs, including improved dyslipidemia, reduced blood pressure, anti-inflammatory, slowed gastric emptying/motility, appetite control, etc. [563,564,565]. Some preclinical studies have indicated that GLP-1RAs may be involved in BAT activation and thermogenesis as well as WAT browning, although firm clinical evidence remains limited [566,567,568,569,570]. A prevailing concern is that adults, especially those with obesity, tend to have significantly reduced amounts of BAT [571]. This has led to skepticism about the feasibility of targeting one of the body’s relatively minor tissue masses to address the broader, systemic challenge of obesity. While WAT browning has undoubtedly expanded the therapeutic landscape by enhancing the body’s thermogenic capacity and activity, the phenomenon of adipose tissue ‘whitening’—the conversion or reversion of BAT and beige adipose tissue to white adipose characteristics—may hamper some of its potential value [572]. In addition, several dose-dependent gastrointestinal adverse events—particularly nausea, vomiting, constipation, and/or diarrhea—have been reported with GLP-1RA use. Thus, these side effects may interfere with necessary dose escalation or even lead to patient discontinuation, thereby limiting GLP-1RA’s overall efficacy [573,574]. With these caveats in mind, some research teams have shifted their attention towards combination drug therapies that can complement the GLP-1RA mechanism while also subsiding some adverse effects [575,576,577]. One promising companion that has been evaluated is the glucose-dependent insulinotropic polypeptide (GIP) [578]. In phase 2 clinical trials, the dual-action agonist LY3298176 was demonstrated to significantly reduce fasting serum glucose and body weight in diabetic subjects [114,579]. Importantly, researchers reported that LY3298176 stayed within an acceptable range for safety and tolerability [114]. Phase 3 clinical trials have supported previous findings, confirming that the GLP-1/GIP dual-receptor agonist not only delivers on its promise but can also outperform some of its single-action competitors [114]. It has been speculated that, aside from its insulinotropic effects, GIP receptor agonist-based therapy may also target WAT health and expansion [580]. This interplay between GIP’s peripheral effects and the already anorexigenic and insulinotropic GLP-1 is becoming widely decreed as the future of drug development for T2DM and other obesity-related disorders [575]. Nonetheless, various loose ends remain. For instance, as previously noted, GLP-1 RAs have been linked to WAT browning, which could potentially conflict with GIP’s role in promoting WAT expansion. Within this context, it is reasonable to question how a dual agonist infringing on the metabolism and function of the same tissue will be able manage two seemingly opposing mechanisms. Another consideration is whether GIP-induced WAT maintenance, even in the context of “healthy” WAT expansion, could begin to paradoxically negate the dual agonist’s initial weight loss effects. Importantly, even patients with metabolically healthy obesity, classified as obesity that lacks overt metabolic symptoms, face many non-metabolic and psychosocial obstacles that are associated with excess WAT preservation [581,582]. Alternatively, it is also possible that GIP effects could be offset by the browning properties of GLP-1, as it has been demonstrated that GIP receptor expression is primarily localized in the tissue’s supporting cells and not directly in the adipocytes [582]. Therefore, careful elucidation of their exact molecular underpinnings, both individually and collectively, is highly warranted. Another emerging area of interest is addressing the sexual dimorphism observed in BAT activation and WAT browning studies. Notably, females appear more susceptible to WAT browning than males [583,584]. Therefore, future drug developments should consider these sex-specific differences to ensure the utmost efficacy and safety for both male and female clients. Taken together, given the multifaceted nature of obesity’s origins, our solutions should be equally as complex, which could involve targeting the activities of multiple adipose tissues and/or pathways to achieve a more comprehensive and effective obesity treatment.

## Data Availability

No new data were created or analyzed in this study. Data sharing is not applicable to this article.

## Abbreviations

The following abbreviations are used in this manuscript:

18F-FTHA	18F-fluoro-6-thia-heptadecanoic acid
AD-Evs	Adipose-derived extracellular vesicles
AGPAT2	1-acylglycerol-3-phosphate-O-acyltransferase 2
AMPK	AMP-activated protein kinase
AOD	Anti-obesity drug
BAT	Brown adipose tissue
BMAT	Bone marrow adipose tissue
BMI	Body mass index
BMP	Bone morphogenetic proteins
C/EBP	CCAAT/enhancer-binding protein
cAMP	Cyclic adenosine monophosphate
CD	Cluster of differentiation
CIDEA	Cell death inducing DFFA like effector a
CIT	Cold-induced thermogenesis Cbp/p300-interacting trans-activator with Glu/Asp-rich carboxy-terminal
Cited 1	domain 1
cMAT	constitutive marrow adipose tissue
CRISPR	Clustered regularly interspaced short palindromic repeats
CT	Computed tomography
DAG	Diacylglycerol
DDS	Drug delivery systems
DGAT	Diacylglycerol Acyltransferase
DIO	Diet-induced obesity
Dio2	Type II iodothyronine deiodinase
DIT	Diet-induced thermogenesis
DNL	De novo lipogenesis
dWAT	Dermal white adipose tissue
FDG	Fluorodeoxyglucose
FFA	Free fatty acid
FGF21	Fibroblast growth factor 21
FNDC5	Fibronectin type III domain-containing protein 5
FO	Fish oil
GIP	Glucose-dependent insulinotropic polypeptide
GLP-1	Glucagon-like peptide-1
GPAT3	Glycerol-3-phosphate acyltransferase 3
HSL	Hormone-sensitive lipase
IGF	Insulin-like growth factor
KO	Knockout
LCFA	Long chain fatty acid
MAT	Marrow adipose tissue
NAD	Nicotinamide adenine dinucleotide
NST	Non-shivering thermogenesis
PAHSA	Palmitic acid hydroxy stearic acids
PAP	PA phosphohydrolase
PET	Positron emission tomography
Pgc1α	Peroxisome proliferator-activated receptor-gamma coactivator 1-alpha
PKA	Protein kinase A
PRDM16	PR Domain Containing 16
RA	Receptor Agonist
rAAV	Recombinant adeno-associated virus
rMAT	regulated marrow adipose tissue
SIRT	Sirtuin
SMAD	Suppressor of mothers against decapentaplegic homolog
SNS	Sympathetic nervous system
T1DM	Type 1 diabetes mellitus
T2DM	Type 2 diabetes mellitus
TAG	Triacylglyerol
TBX1	T-box 1
TGF-β	Transforming growth factor-β
TMEM26	Transmembrane protein 26
UCP1	Uncoupling protein 1
WAT	White adipose tissue
YAT	Yellow adipose tissue
β3-AR	β3-adrenoceptor
